# Identification of therapeutic targets of the hijacked super-enhancer complex in *EVI1*-rearranged leukemia

**DOI:** 10.1038/s41375-021-01235-z

**Published:** 2021-04-28

**Authors:** Sandra Kiehlmeier, Mahmoud-Reza Rafiee, Ali Bakr, Jagoda Mika, Sabrina Kruse, Judith Müller, Sabrina Schweiggert, Carl Herrmann, Gianluca Sigismondo, Peter Schmezer, Jeroen Krijgsveld, Stefan Gröschel

**Affiliations:** 1 Molecular Leukemogenesis, German Cancer Research Center, Heidelberg, Germany; 2 Bioinformatics and Computational Biology Laboratory, The Francis Crick Institute, London, United Kingdom; 3 Proteomics of Stem Cells and Cancer, German Cancer Research Center, Heidelberg, Germany; 4 Cancer Epigenomics, German Cancer Research Center, Heidelberg, Germany; 5 Health Data Science Unit, Medical Faculty Heidelberg and BioQuant, Heidelberg, Germany; 6 Heidelberg University, Medical Faculty, Heidelberg, Germany; 7 Internal Medicine V, Heidelberg University Hospital, Heidelberg, Germany; 8 Oncology Center Worms, Worms, Germany

**Keywords:** Cancer epigenetics, Acute myeloid leukaemia

## Abstract

Deregulation of the *EVI1* proto-oncogene by the *GATA2* distal hematopoietic enhancer (*G2DHE*) is a key event in high-risk acute myeloid leukemia carrying 3q21q26 aberrations (3q-AML). Upon chromosomal rearrangement, *G2DHE* acquires characteristics of a super-enhancer and causes overexpression of *EVI1* at 3q26.2. However, the transcription factor (TF) complex of *G2DHE* remains poorly characterized. The aim of this study was to unravel key components of *G2DHE*-bound TFs involved in the deregulation of *EVI1*. We have identified several CEBPA and RUNX1 binding sites to be enriched and critical for *G2DHE* function in 3q-AML cells. Using ChIP-SICAP (ChIP followed by selective isolation of chromatin-associated proteins), a panel of chromatin interactors of RUNX1 and CEBPA were detected in 3q-AML, including PARP1 and IKZF1. PARP1 inhibition (PARPi) caused a reduction of *EVI1* expression and a decrease in *EVI1*–*G2DHE* interaction frequency, highlighting the involvement of PARP1 in oncogenic super-enhancer formation. Furthermore, 3q-AML cells were highly sensitive to PARPi and displayed morphological changes with higher rates of differentiation and apoptosis as well as depletion of CD34 + cells. In summary, integrative analysis of the 3q-AML super-enhancer complex identified CEBPA and RUNX1 associated proteins and nominated PARP1 as a potential new therapeutic target in *EVI1* + 3q-AML.

## Introduction

Aberrant expression of the zinc finger transcription factor (TF) Ecotropic Viral Integration Site 1 (EVI1) is a potent oncogenic event involved in the pathogenesis of high-risk hematopoietic neoplasms [1, 2]. *EVI1* is transcribed from the *MECOM* locus on chromosome 3q26.2, which also encodes the Myelodysplasia Syndrome-associated Protein 1 (*MDS1*) and the longer splice form *MDS1-EVI1* [3, 4]. EVI1 and its isoforms are part of the PRDI-BF1 (positive regulatory domain I-binding factor 1) and RIZ1 (retinoblastoma protein-interacting zinc finger gene 1) homology domain (PRDM) family of epigenetic remodelers, with EVI1 also being known as PRDM3 [5, 6]. Although its exact molecular function is still poorly understood, EVI1 plays an essential role in a range of transcriptional regulatory networks important for stem cell maintenance and chromatin remodeling [7–9]. EVI1 is known as the most oncogenic isoform and widely studied in leukemia, while MDS1-EVI1 is thought to function as a tumor suppressor [10, 11]. Imbalanced expression of *MECOM*-transcribed isoforms is often the consequence of (i) chromosomal rearrangements leading either to *EVI1* gene fusions (e.g., *AML1-EVI1*) [12]; (ii) *MECOM* locus amplifications as found with high frequency in high-grade serous ovarian cancer [13]; (iii) aberrant promoter activation [14]; or (iv) displacement of regulatory DNA elements into the *EVI1* locus, the latter being a hallmark of 3q26.2/*MECOM* rearranged acute myeloid leukemia (AML) [15, 16].

In AML, 3q26.2 rearrangements targeting the *MECOM* locus typically abrogate expression of both the long *MDS1-EVI1* isoform and, coincidentally, expression of other key myeloid regulators, such as *GATA2* or *MYC*, while transcription of *EVI1* becomes excessively increased [15–18]. Similar to *EVI1* overexpression caused by inv(3)/t(3;3) in AML, also other PRDM family members are found upregulated in leukemias as for example *PRDM16* (*MEL1*) in t(1;3)/*MEL1* rearranged AML [19]. The underlying mechanism of forced *EVI1* or *MEL1* transcription and co-silencing of *GATA2* in 3q-rearranged AML is the repositioning of a *GATA2* distal hematopoietic enhancer (*G2DHE*) into the vicinity of these *EVI1* homologs [16, 18, 20]. Relocation of non-coding regulatory DNA elements has recently been coined as enhancer hijacking, and we and others have previously shown that the specific *G2DHE* element in 3q-rearranged AML is a monoallelic super-enhancer formed on the oncogenic *EVI1* allele [15–17, 21].

To date, little is known about the molecular composition of the TFs occupying this oncogenic super-enhancer that regulates *EVI1*, obscuring the identification of novel therapeutic targets that are needed to treat this almost invariably fatal type of AML [22]. In order to better understand the regulation of *EVI1* in 3q-rearranged AML, we have performed proteomic analyses of chromatin-bound TFs known to be present at *G2DHE*, such as CEBPA and RUNX1, and have identified colocalizing complex members of the super-enhancer. Among these, we have focused on translational targets for further characterization in vitro as these may have potential therapeutic significance.

## Methods

### PARP1 inhibitors

Olaparib (AZD2281; Selleckchem, Houston, TX, USA) and talazoparib (T6253; TargetMol, Wellesley Hills, MA, USA) were resuspended in DMSO at a concentration of 100 mM or 200 mM, respectively.

### Dual-luciferase reporter assay

The pGL3 basic vector (Promega, Madison, WI, USA) and the pRL-SV40 *Renilla* vector (Promega, Madison, WI, USA) were used in all luciferase assays. The pGL3 vector containing the *EVI1* promoter was a gift from K. Mitani, Dokkyo Medical University School of Medicine, Kitakobayashi, Japan. Different *G2DHE* variants were cloned into this vector using the restriction enzymes BamHI and SalI (Table S1). The pGL3 vector containing the *EVI1* promoter and full-length *G2DHE* was described previously [16]. Site-directed mutagenesis was carried out using the QuikChangeII XL kit (Agilent Technologies, Santa Clara, CA, USA) (Table S2). Mutated constructs were re-cloned into a fresh backbone vector. Cells were seeded at a density of 0.5 × 10^6^ cells/mL and transiently transfected with X-tremeGENE HP DNA (Roche, Basel, Switzerland) transfection reagent according to the manufacturer’s protocol. Per 0.5 × 10^6^ cells 100 ng of pRL-SV40 and 900 ng of the full-length *G2DHE* constructs were used. Equimolar amounts were used for shorter pGL3 constructs. Cells were harvested 48 h after transfection. Dual-luciferase assays were performed using the Dual-Luciferase Reporter Assay System (Promega, Madison, WI, USA) according to the manufacturer’s protocol on a Victor X3 plate reader (Perkin Elmer, Waltham, MA, USA). Luciferase signal was normalized to *Renill*a signal.

### In silico prediction of TF binding sites

The sequence of the *G2DHE* core was fed into the online tools JASPAR2016 (JASPAR database, http://jaspar2016.genereg.net/cgi-bin/jaspar_db.pl?rm=browse&db=core&tax_group=vertebrates) and Alggen Promo (TRANSFAC database version 8.3, http://alggen.lsi.upc.es/cgi-bin/promo_v3/promo/promoinit.cgi?dirDB=TF_8.3) [23–25]. Matrices for selection of important myeloid TFs were chosen and the sequence was scanned with a threshold of 80% relative profile score for JASPAR2016 or 15% dissimilarity score for Alggen Promo. Predicted TF binding sites (TFBS) were checked for conservation between species. Mutations were aimed to have a relative similarity score of less than 70% with an ideal value below 50–60% (JASPAR2016) or more than 15% dissimilarity score (Alggen Promo).

### Lentiviral constructs

Short hairpin RNA (shRNA) knockdown experiments were carried out with the optimized microRNA-30 backbone element (miR-E) for CEBPA, RUNX1 and PARP1 [26]. The SGEP vector was a gift from J. Zuber (Research Institute of Molecular Pathology, Vienna, Austria; Addgene plasmid #111170). Genome-wide sensor-based shRNA predictions were used for the choice of the target sequence [26]. 97-mer Ultramer DNA Oligos (Table S3) were ordered from Integrated DNA Technologies and cloned into the SGEP vector as described previously (Table S1) [26].

For *EVI1* knockdown, pLKO.1-Puro (Bob Weinberg, Addgene plasmid #8453) harboring shRNAs targeting *EVI1* (TRCN0000002532, TRCN0000002531) or a commercial non-targeting control (SHC002, Sigma-Aldrich, St. Louis, MO, USA) were used.

The *EVI1* ORF was cloned into pLenti-CMV-Puro-DEST (w118-1) (Eric Campeau & Paul Kaufman, Addgene plasmid #17452). pLenti-CMV-Puro containing the coding sequence for 1xFlag was used as empty vector control.

A detailed transduction protocol is included in the supplemental methods.

### ChIP-SICAP

Chromatin immunoprecipitation with selective isolation of chromatin-associated proteins (ChIP-SICAP) was conducted as previously described with few modifications described in the supplement [27, 28]. In brief, cells fixed with formaldehyde were lysed and the nuclear fraction was extracted. Chromatin was sheared using sonication and incubated with antibodies directed against the respective bait proteins or an IgG control. Protein complexes were purified using magnetic beads. Following immunoprecipitation, the DNA bound to the protein complexes was biotinylated and chromatin-associated complexes were purified using streptavidin beads. Eluted proteins and DNA were used for mass spectrometric analysis and qPCR, respectively.

### DNA streptavidin pull-down

Nuclear lysate was incubated with PCR-generated (Table S1), biotinylated DNA probes. DNA probes and associated proteins were purified using streptavidin magnetic beads (S1420S; New England Biolabs, Frankfurt am Main, Germany) and analyzed by SDS-PAGE and western blot. 30 µg of nuclear lysate and 30 µL of the last washing step were used as controls. A detailed protocol is included in the supplemental methods.

### Flow cytometry

Apoptosis staining was conducted using the FITC Annexin V Apoptosis Detection Kit I (BD Bioscience, San Jose, CA, USA). Staining for differentiation markers was performed using the following antibodies directed against cell surface markers: CD34−PerCPCy5.5 (#343611; Biolegend, San Diego, CA, USA), CD11b−APC (#301309; BioLegend, San Diego, CA, USA), CD14−APCH7 (MϕP9; BD Biosciences, San Jose, CA, USA). For intracellular staining, either one of the following antibodies were used: cMPO-FITC (sc-51741 FITC; Santa Cruz Biotechnology, Dallas, TX, USA) or γH2AX-Alexa488 (560445, BD Biosciences, San Jose, CA, USA). A detailed staining protocol is attached in the Supplementary methods.

## Results

### The hijacked *G2DHE* harbors two conserved sequence modules enriched for RUNX1, CEBPA, and MYB motifs

**Fig. 1:**
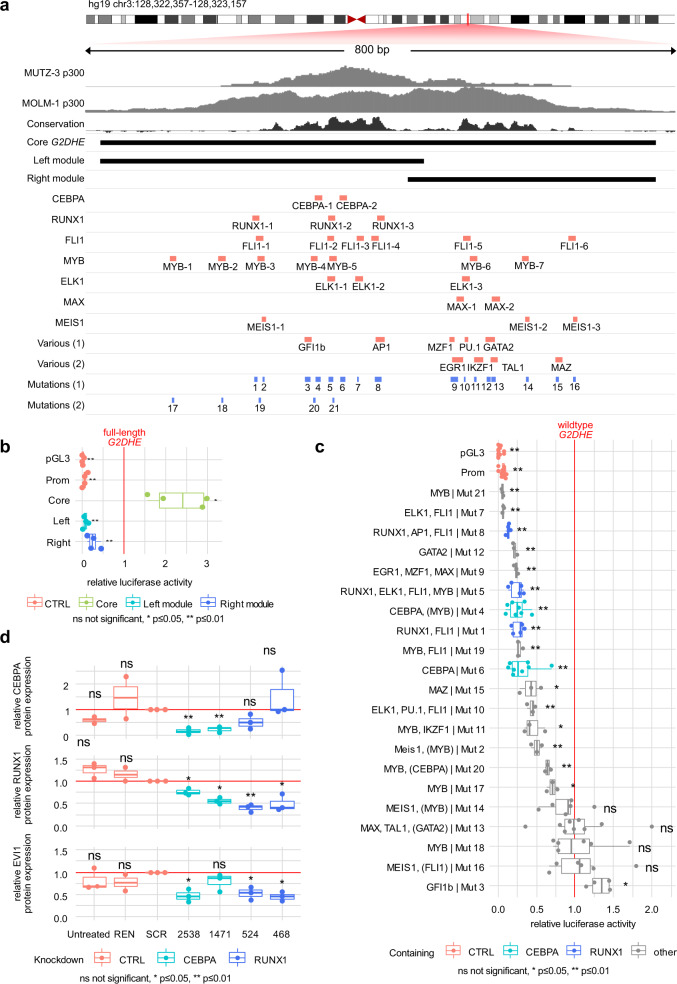
CEBPA and RUNX1 function as transcriptional coactivators of *G2DHE* in vitro in 3q-rearranged AML. **a** Schematic overview of the transcription factor binding sites identified with the JASPAR2016 and TRANSFAC databases as well as the respective mutations and truncated enhancer fragments in relation to the previously described p300 chromatin binding peaks in MUTZ-3 and MOLM-1 cells and the conservation across species (SiPhy rate 10 mer) [16, 54]. **b**, **c** Luciferase reporter assay in HNT-34. Cells were co-transfected with the luciferase reporter plasmid pGL3 and the *Renilla* control plasmid. pGL3 empty vector (pGL3), pGL3 vector containing the *EVI1* Promoter (Prom), and pGL3 vector containing the *EVI1* promoter and *G2DHE* were used as controls (CTRL). Luciferase signal was normalized to *Renilla* signal. The relative luciferase signal was further normalized to the signal of full-length, wildtype *G2DHE* (red line). Statistical significance was calculated using two-sided one-sample *t*-tests. **b** shows the activity of the truncated enhancer fragments (*n* = 4) and **c** shows the activity of enhancer mutants (*n* ≥ 3). TFBS in parentheses are in close proximity to the mutations without being affected by them. **d** Protein expression quantification following *CEBPA* and *RUNX1* knockdown in MUTZ-3 cells using miR-E constructs (*n* = 2 for REN, *n* = 3 for all other samples). Cells were lentivirally transduced and selected with puromycin. Samples were harvested on day 3 of puromycin selection, and protein levels were analyzed by western blot using antibodies against CEBPA, RUNX1, and EVI1. Western blot signal of the proteins of interest was normalized to the respective loading control and to the scrambled control (SCR). A miR-E construct targeting *Renilla* (REN) and untreated parental cells serve as additional non-targeting controls. Statistical significance was calculated using two-sided one-sample *t*-tests.

Hijacking of *G2DHE* is the underlying molecular event in the pathogenesis of inv(3)/t(3;3) AML, as we and others have previously described [15, 16]. We aimed to better understand the molecular basis of the enhancer function in this AML subset by studying the complex of its associated TFs. The core p300-binding portion of *G2DHE* consists of a highly conserved bimodular sequence structure (Fig. 1a). TFBS prediction analyses showed predominant enrichment of MYB, RUNX1, and CEBPA motifs in the left (centromeric) part, while other prominent myeloid TFs, such as PU.1, GATA2, TAL1, and IKZF1 motifs clustered in the right (telomeric) region. In *EVI1*-promoter-luciferase reporter studies, a 755 bp region of the core *G2DHE* was sufficient to induce reporter gene activity, whereas neither left and right module alone exhibited transactivating potential in the t(3;3) HNT-34 cells (Fig. 1b). In order to assess the importance of individual TFBS in vitro, we generated inactivating mutants of the identified TFBS and studied their impact on reporter gene induction (Figs. 1a and S1a). The majority of mutations (16/21) led to reduced enhancer reporter activity in HNT-34 (Fig. 1c), and integration of data from three inv(3)/t(3;3) cell lines (MOLM-1, MUTZ-3, HNT-34) showed consistent downregulation when TFBS of CEBPA, FLI1, MYB, and RUNX1 were mutated (Fig. S1b). For validation of candidate TFs used in further proteomic studies, we chose RUNX1 and CEBPA, since (i) both TFs are pioneer TFs and key myeloid transcriptional regulators expressed at sufficiently high levels in inv(3)/t(3;3) AML allowing for subsequent protein capture experiments (Fig. S2a, b); (ii) RUNX1 has been implicated in the regulation of *EVI1* previously [29]; and (iii) CEBPA mutations are mutually exclusive with inv(3)/t(3;3) in AML [1, 30]. Furthermore, EVI1 expression was downregulated following global miR-E -mediated knockdown of CEBPA and RUNX1 in inv(3) AML cells (Fig. 1d), suggesting functional importance of these myeloid TFs in maintaining EVI1 expression by either direct or indirect effects.

### Enhancer-bound CEBPA and RUNX1 colocalize with IKZF1 and PARP1

**Fig. 2:**
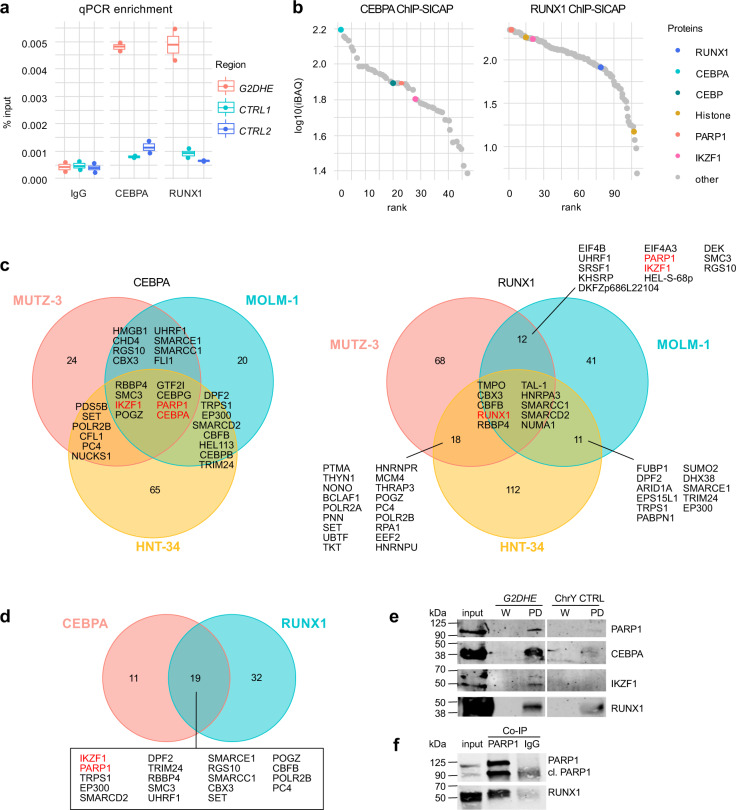
CEBPA and RUNX1 form a complex with IKZF1 and PARP1 at *G2DHE* in 3q-rearranged AML. ChIP-SICAP was performed in three different 3q-rearranged cell lines using antibodies against CEBPA and RUNX1 or an unspecific IgG control. Experiments were carried out in duplicates (*n* = 2). **a**, **b** show exemplary results for the cell line MUTZ-3. **a** The DNA fraction of the ChIP-SICAP experiment was used for enrichment quantification of the *G2DHE* region by qPCR compared to two unrelated control regions (CTRL). **b** The protein fraction of the ChIP-SICAP experiments was analyzed by mass spectrometry to identify chromatin-bound interactors of RUNX1 and CEBPA. Proteins enriched in the CEBPA- or RUNX1-captured samples over the IgG controls in both MUTZ-3 replicates were ranked according to their iBAQ intensity. **c** Overlap of the proteins identified in different cell lines. **d** Overlap of proteins that were identified in at least two cell lines per bait. **e** DNA-streptavidin pull-down (PD) of CEBPA, RUNX1, IKZF1, and PARP1. Nuclear lysate of MUTZ-3 cells was incubated with biotinylated DNA probes containing the *G2DHE* sequence or a deserted control region from chromosome Y (ChrY CTRL). Proteins associated with the DNA probes were purified and analyzed by western blot using antibodies against CEBPA, RUNX1, IKZF1, and PARP1. Input and an aliquot of the last wash steps (W) served as control for the PD samples. **f** Co-immunoprecipitation of PARP1 and RUNX1. Total cell lysate of MUTZ-3 was used for immunoprecipitation either with a PARP1 antibody or a control IgG. 75 µg of lysate was used as input control. Western blot was performed using antibodies directed against PARP1 and RUNX1.

**Fig. 3:**
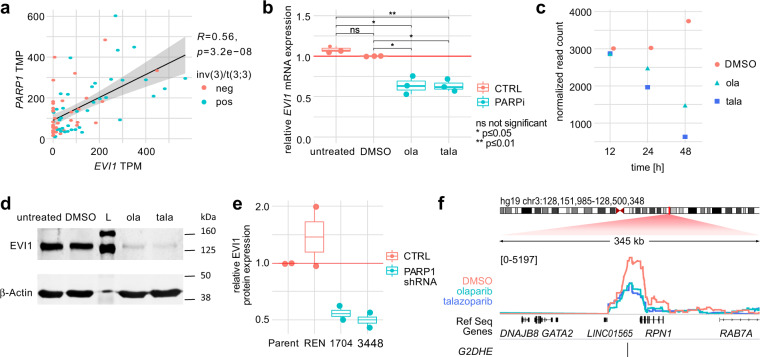
PARP1 inhibition leads to lower *EVI1* expression and decreased interaction frequency between *G2DHE* and the *EVI1* promoter. **a** Correlation of *EVI1* and *PARP1* normalized transcripts per million (TPM) expression levels. Values were measured by RNA-Seq in human primary AML patient samples (*n* = 73) and cell lines (*n* = 9) as described previously [16]. Shown is the Pearson correlation coefficient (*R*) with p-value (*p*). **b** EVI1 mRNA expression after PARPi. Cells were treated with 10 µM olaparib (ola), 1 µM talazoparib (tala), or DMSO or were left untreated. *EVI1* mRNA expression was analyzed by qPCR following PARPi treatment for 24 h (*n* = 3). Results are normalized to the housekeeping gene *HMBS* and the DMSO sample. Statistical significance was calculated using two-sided two-sample t-tests. **c** Normalized read counts of MUTZ-3 treated with DMSO, 10 µM olaparib (ola), or 1 µM talazoparib (tala) across multiple time points as determined by RNA-Seq. **d** Western blot analysis of EVI1 protein levels in MUTZ-3 cells after 24 h PARPi with 10 µM olaparib (ola) or 1 µM talazoparib (tala). The protein ladder is marked with L. **e** Protein expression quantification following *PARP1* knockdown in MUTZ-3 cells using miR-E constructs (*n* = 2). Cells were lentivirally transduced and selected with puromycin. Samples were harvested on day 3 of puromycin selection, and protein levels were analyzed by western blot using antibodies against PARP1 and EVI1. Western blot signal of the proteins of interest was normalized to β-Actin and to the parental control. A miR-E construct targeting *Renilla* (REN) and untreated parental cells served as non-targeting controls. **f** Semiquantitative analysis of the local chromatin interaction profile of the *EVI1* promoter at the 3q21 *G2DHE* location as determined by 4C-Seq in MUTZ-3 cells following treatment with 10 µM olaparib, 1 µM talazoparib, or DMSO for 24 h. The 4 C signal is measured by calculation of a sliding window average (running mean) of the normalized read counts (window size: 21 fragment ends). The 4 C tracks were group autoscaled and the numbers indicate the data range.

To identify proteins that colocalize with CEBPA and RUNX1 in enhancer-bound TF complexes and that are potential therapeutic targets in 3q-rearranged AML, we performed ChIP-SICAP, a method that allows for the isolation of protein complexes in their chromatin-bound state by using specific bait proteins [27]. CEBPA and RUNX1 served as baits, and their locus-specific association with *G2DHE* was confirmed by ChIP-Seq (Fig. S2c) and qPCR on DNA purified after ChIP-SICAP in the three inv(3)/t(3;3) AML cell lines MUTZ-3, HNT-34, and MOLM-1 (Figs. 2a and S2d). Next, the protein fractions of genomic loci captured with the bait proteins were analyzed by quantitative mass spectrometry (Figs. 2b, S2e–f, and File S1). Successful enrichment of chromatin-associated proteins was evidenced by the detection of histones and by nuclear localization of identified proteins of at least 76% of all captured proteins, as well as by chromatin binding and transcription as some of the most significantly enriched biological processes and molecular functions in all cell lines using both baits according to Gene Ontology (GO) analysis (Figs. 2b and 3a, b). Following this analysis, the majority of all identified proteins were categorized as potential true positives (Fig. S3c). We detected 46 (MUTZ-3), 44 (MOLM-1), and 87 (HNT-34) proteins when using CEBPA as bait, and 108 (MUTZ-3), 74 (MOLM-1), and 151 (HNT-34) proteins following RUNX1 capture (Fig. 2c). Already known interactors of the bait proteins CEBPA (17% of all identified ChIP-SICAP proteins) and RUNX1 (33% of all identified ChIP-SICAP proteins) were detected using Biological General Repository for Interaction Datasets (BioGRID) analyses (Fig. S3a, b and Table S4). When comparing different cell lines, 30 proteins were identified in at least two cell lines using CEBPA as bait, and 51 for RUNX1 (Fig. 2d). Within the CEBPA and RUNX1 datasets, we found a total of 19 proteins to be enriched for both bait proteins in at least two inv(3)/t(3;3) cell lines (Fig. 2d). These included previously described enhancer-associated proteins, such as the histone acetyltransferase p300 (EP300) that initially led us to the identification of the *G2DHE* [16]. Furthermore, among the recurrently detected candidates were Ikaros family zinc finger 1 (IKZF1), a TF previously described in inv(3)/t(3;3) AML, and poly(ADP-ribose) polymerase 1 (PARP1) (Fig. 2c, d) [31].

Since ChIP-SICAP represents a global interrogation of interactors with bait-bound chromatin loci genome-wide, we subsequently tested binding of IKZF1 and PARP1 to *G2DHE* sequence probes in nuclear lysates of MUTZ-3 cells (Fig. 2e). Compared to an unrelated control probe covering a desert chromatin locus on chromosome Y, both CEBPA and RUNX1 bait proteins as well as IKZF1 and PARP1 were detected (Fig. 2e). This confirms that the proteins identified with ChIP-SICAP can associate with the *G2DHE* sequence in vitro and thus may represent rational targets for further validation. Moreover, interaction of PARP1 and RUNX1 was further validated by co-immunoprecipitation (Fig. 2f). Interaction of CEBPA and PARP1 has been previously demonstrated in prostate cancer, however, we could not validate their interaction in AML cells due to overlap with unspecific antibody/bead complex signals [32].

### PARP1 inhibition reduces *EVI1* expression

Besides its canonical role in DNA repair, PARP1 has been found to have important functions in cooperating with cellular key identity TFs to organize chromatin structures, bind enhancers, and create chromatin environments permissive of transcription in embryonic stem cell and neural differentiation [33, 34]. We thus hypothesized that PARP1 might be integral to the establishment of *G2DHE* into an oncogenic super-enhancer on the rearranged 3q allele and necessary to maintain *EVI1* expression in inv(3)/t(3;3) AML. *EVI1* and *PARP1* mRNA expression was significantly correlated as determined by RNA-Seq analysis of previously described primary AML patient samples and cell lines (Fig. 3a and File S2) [16], suggesting coregulation of the two genes in AML. It has recently been shown that PARP1-mediated establishment of activating enhancer-promoter interactions are dependent on the catalytic activity of PARP1 [35]. To assess whether PARP1 activity is required for *EVI1* transcription, we treated MUTZ-3 cells with the PARP1 inhibitors olaparib (10 µM) and talazoparib (1 µM) (Fig. 3b), both being potent inhibitors of the catalytic activity of PARP1, while talazoparib also causes trapping of PARP1 on chromatin. For both compounds, we found a significant reduction in *EVI1* mRNA expression following PARP1 inhibition (PARPi) as compared to vehicle control after 24 h. RNA-Seq confirmed the decrease of *EVI1* expression in a time-dependent manner upon PARPi (Fig. 3c). This effect was evident even more strongly on protein level (Figs. 3d and S4a). Additionally, similar effects could be observed upon shRNA-mediated *PARP1* knockdown (Figs. 3e and S4b). In order to determine if *EVI1* downregulation was accompanied by reduced chromatin interaction between the *EVI1* promoter and the rearranged *G2DHE* upon PARPi, we performed 4C-Seq in MUTZ-3 cells after 24 h of PARPi treatment. Using the *EVI1* promoter as viewpoint, 4C-Seq comparative analysis revealed a decreased interaction frequency between the rearranged *G2DHE* and the *EVI1* promoter in response to PARPi, corroborating previous reports on the necessity of PARP1 occupancy of certain enhancers in long-range gene promoter interactions (Fig. 3f) [35–37].

### PARP1 inhibition affects cell survival and morphology

**Fig. 4:**
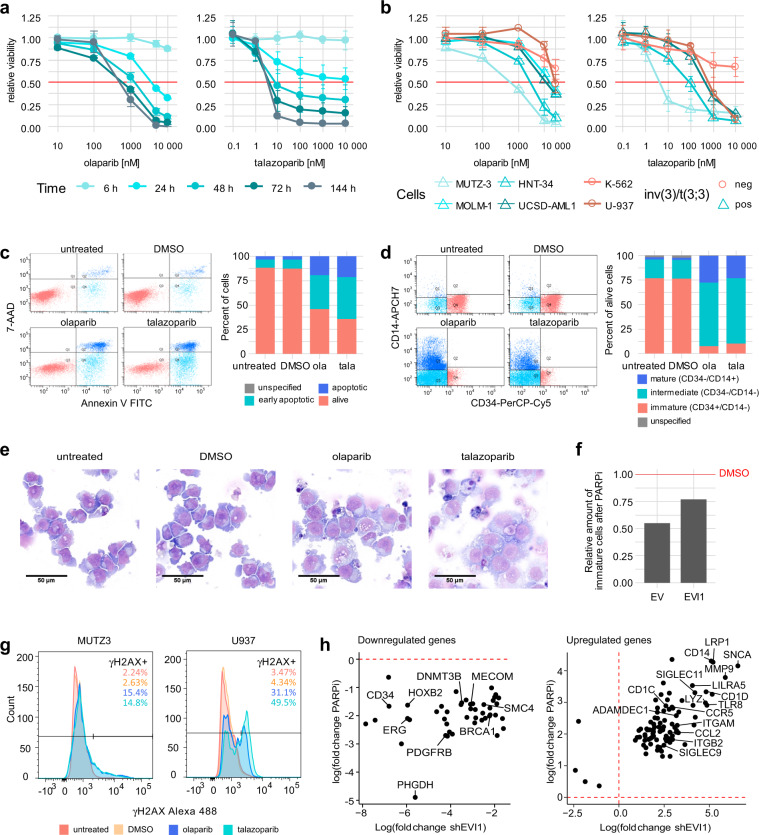
PARP1 inhibition (PARPi) causes phenotypical changes and apoptosis of MUTZ-3 cells. **a**, **b** Sensitivity of different cell lines to PARPi. Cells were treated with the indicated amounts of olaparib or talazoparib. Shown is the mean ± SD (*n* = 3). Metabolic activity was measured as an indicator of cell viability by CellTiter-Glo assay. Values were normalized to those of the 0 h time point and to the DMSO control of each time point. **a** Viability of MUTZ-3 cells across multiple time points. **b** Comparison of several 3q-rearranged and non-rearranged cell lines at 72 h. **c**–**e** Effects of PARPi treatment in MUTZ-3 cells. Cells were treated with 10 µM olaparib (ola), 1 µM talazoparib (tala), or DMSO or were left untreated for 24 h. **c** Apoptosis staining with Annexin-V and 7-AAD and flow cytometric analysis. The graphs show one representative replicate. **d** Flow cytometric analysis of differentiation markers. Cells were gated for the live population. The graphs show one representative replicate. **e** Representative images of May-Grünwald-Giemsa staining of control and PARPi treated MUTZ-3 cells. **f** *EVI1* overexpression partially rescues loss of immature cells after PARPi. MUTZ-3 cells were transduced using a lentiviral expression vector containing *EVI1* or the corresponding empty vector (EV) control. After selection with puromycin, cells were treated with 10 µM olaparib or DMSO for 24 h. Differentiation markers were assessed by flow cytometry. Cells were gated for the live cell population. The amount of immature (CD34 + /CD14−) cell population of PARPi treated cells was normalized to the DMSO control. The graph shows one representative replicate. **g** DNA damage response after PARPi in MUTZ-3 and U-937. Intracellular γH2AX levels of untreated cells or cells treated with DMSO, 10 µM olaparib, or 1 µM talazoparib were assessed by flow cytometric analysis. Cells were gated for the single cell population. **h** Commonly downregulated (left) and upregulated (right) genes by PARPi and *EVI1* knockdown as identified by RNA-Seq. Genes included in cluster 3 of the PARPi data were compared to the genes deregulated by *EVI1* knockdown to determine the genes downregulated by both conditions. Upregulated genes in cluster 4 of the PARPi RNA-Seq data were determined accordingly. Fold change of gene expression under treatment conditions (PARPi or shEVI1, respectively) over the control (DMSO or non-targeting control, respectively) is shown. Exemplary genes are annotated.

Olaparib and talazoparib treatment of MUTZ-3 cells led to a substantial reduction in cell viability (Fig. 4a). The growth inhibitory effect of PARPi was also apparent in various other 3q-rearranged AML cells lines MOLM-1, HNT-34, and UCSD-AML1 in a concentration- and time-dependent manner (Figs. 4b and S5) with higher efficacy of talazoparib compared to olaparib, while the non-3q-rearranged AML cell lines U-937 and K-562 showed a tendency to be less sensitive towards the PARPi treatment. When treating MUTZ-3 cells with 1 µM talazoparib or 10 µM olaparib, the reduction of cell viability of AML cells went along with a higher percentage of apoptotic cells as shown by Annexin-V/7-AAD staining in flow cytometric analysis (Fig. 4c). Similar to effects previously seen upon *EVI1* downregulation induced by *G2DHE* deletion, immunophenotyping of PARPi treated inv(3) MUTZ-3 cells showed a loss of CD34 expression along with an increase in myelomonocytic differentiation as measured by CD14 expression (intermediate: CD34−/CD14− and mature: CD34−, CD14+), whereas the immature progenitor cells (CD34+/CD14−) were almost completely lost in comparison to the controls (Fig. 4d). Furthermore, cell morphology assessment of PARPi treated cells using May–Grünwald–Giemsa staining confirmed morphological changes with bigger cytoplasm, more vacuolization, and appearance of monocyte-like cells after treatment with talazoparib or olaparib compared to the DMSO vehicle control and untreated cells after 24 h (Fig. 4e).

Next, we investigated whether the phenotype observed after PARPi was primarily dependent on epigenetic downregulation of *EVI1* transcription or rather secondary to PARP1-specific functional effects, such as induction of DNA damage. Forced expression of *EVI1* partially rescued the loss of immature progenitor cells upon PARPi treatment (Fig. 4f), suggesting a direct interaction between *EVI1* and PARP1. Furthermore, DNA damage was monitored by assessing intracellular γH2AX levels (Fig. 4g). PARPi induced a large increase of γH2AX in the 3q-negative cell line U-937, however, in the 3q-rearranged cell line MUTZ-3 only a low to moderate increase was observable.

RNA-Seq analysis of PARPi treated MUTZ-3 cells revealed 4 distinct clusters among the 1 500 most differentially regulated genes (Fig. S6a and File S3). Of those, cluster 2 (159 genes) and cluster 4 (756 genes) showed higher gene expression after PARPi, whereas cluster 3 (454 genes) was downregulated. Pathway enrichment analysis of the different clusters (Fig. S6b–e) identified cell cycle control and DNA repair-related pathways to be enriched among genes downregulated after PARPi treatment (Fig. S6d). Neutrophil degranulation, interferon signaling, and other immune system-related pathways were enriched among upregulated genes (Fig. S6e), which supports the observation of a differentiation phenotype of inv(3) AML cells following PARPi treatment (Fig. 4d–e). Correlation of RNA-Seq data upon either PARPi (cluster 3) or *EVI1* knockdown (File S3) revealed that 46 genes were commonly downregulated between the two conditions, including myeloid regulators *CD34*, *ERG*, *HOXB2*, and *EVI1* itself (Figs. 4h and S7a, b). 91 genes were found to be commonly upregulated between the two conditions when using PARPi cluster 4 for comparison with the *EVI1* knockdown (Figs. 4h and S7c, d), including genes involved in immune cell maturation like *CD14*.

Overall, these data indicate that DNA damage and impaired DNA damage response do not appear to be the primary cause of PARPi sensitivity and subsequent *EVI1* transcriptional disturbance in the 3q-rearranged MUTZ-3 cells.

## Discussion

The context-dependent oncogenic functions of EVI1 family members have increasingly been recognized in multiple types of cancer, although the mechanisms of oncogenic transformation caused by EVI1 are complex and incompletely understood [11, 13, 38]. Previous reports support the notion that the tight interplay between deregulated EVI1 and simultaneous disturbances in the dosage of other myeloid TFs, such as GATA2, is linked to the development and maintenance of therapy-resistant AML with 3q26.2/*EVI1* rearrangements [16–18, 20]. From these studies, *G2DHE* emerged as a common regulatory node between these TFs and as a key regulator of hematopoiesis with leukemogenic consequences when misplaced [15–18, 20, 29]. However, the molecular architecture and functional characteristics of *G2DHE* have been little understood, particularly with respect to *EVI1*-rearranged AML. This prompted us to investigate the TF composition of *G2DHE* and potential interactors.

Our studies provide molecular and functional evidence of the TF composition of *G2DHE* in inv(3)/t(3;3) AML that may render it a therapeutic liability for this disease category. The interplay and additive effect of those TFBS is integral to *G2DHE* function as both modules are required for maximum enhancer activity. Among the most recurrent and functionally important TFs in reporter studies of *G2DHE* in vitro were RUNX1, MYB, CEBPA, IKZF1, FLI1, ELK1, and GATA2 itself, which play key roles in hematopoiesis [39–41]. In line with our observations, RUNX1, MYB, and ELK1 have previously been implicated in the regulation of *EVI1* transcription in AML cells by studies of the minimal *EVI1* promoter of the *MECOM* locus, which is the target of the hijacked *G2DHE* in 3q-rearranged AML [29]. Although, *CEBPA* has been reported to be negatively regulated by EVI1, we have observed measurably high expression of CEBPA in inv(3)/t(3;3) cell lines (Fig. S2b), clear binding of CEBPA at *G2DHE* in inv(3)/t(3;3) cells, and activating potential in reporter experiments [42]. The presence of RUNX1 and CEBPA binding at *G2DHE* allowed targeted chromatin-capture and thereby identification of novel interacting partners. A limitation of ChIP-SICAP is its lack of targeted identification of locus-specific interactions, thus it was not possible to discriminate between TF complexes of the orthotopic and rearranged *G2DHE* alleles. However, histone ChIP-Seq data point to the generation of a super-enhancer accessible for TFs only on the rearranged 3q21 allele [16].

ChIP-SICAP was used to identify a panel of CEBPA and RUNX1 interactors. Association of the two hits PARP1 and IKZF1 was confirmed by an in vitro pull-down approach (Fig. 2e); however, ChIP(-Seq) of these two hits in our 3q-rearranged model cell lines proved not to be feasible.

The mapping of IKZF1 DNA binding sites in *G2DHE* impacting *EVI1* promoter activity and chromatin co-occupancy with RUNX1 and CEBPA as found in ChIP-SICAP experiments implicate an interaction of IKZF1 with EVI1/GATA2 pathways, which was previously indicated by a study investigating the mutational landscape of *EVI1*-rearranged AML [31]. We have observed a slight impact on *EVI1* expression, but no reduced viability of inv(3)/t(3;3) AML cell lines in response to lenalidomide treatment (data not shown), which causes selective degradation of IKZF1 [43]. Notwithstanding, the use of lenalidomide has been suggested as a possible adjunct to hypomethylating agents in the treatment of inv(3)/t(3;3) AML patients [44].

PARP1 was identified in ChIP-SICAP experiments using both *G2DHE* baits RUNX1 and CEBPA in different 3q-rearranged cell lines. Inhibition of PARP1 catalytic activity and *PARP1* knockdown led to downregulation of *EVI1*. RNA-Seq analysis showed that more genes were upregulated than downregulated after PARPi treatment (Fig. S6a). Hence, it appears that instead of causing a global decrease of transcription, the downregulation of *EVI1* does not appear to be unspecific. Further analysis of cells treated with PARPi also showed a profound growth arrest of inv(3) AML cells, and increased differentiation, which can be partially rescued by overexpression of *EVI1*. This differentiation pattern was similar to that observed after *EVI1* knockdown, BRD4 inhibition, or *G2DHE* deletion previously [16]. Comparative RNA-Seq analysis following PARPi and *EVI1* knockdown confirmed induction of differentiation gene expression patterns and revealed a subset of coregulated genes. Interestingly, one of the strongest negatively regulated gene both in PARPi and *EVI1* knockdown in inv(3) AML cells was *PHGDH*, a serine biosynthesis enzyme implicated as a critical metabolic regulator necessary for propagation of leukemia cells and other cancers [45–47].

Previous studies showed that PARP1 can exert its positive gene regulatory function by poly(ADP-ribosyl)ation (PARylation) of histones and the histone-associated protein DEK (Fig. 2b) and the histone deacetylase SIRT, leading to chromatin decompaction, binding of Mediator coregulatory complex, and initiation of transcription [48–50]. Here, PARPi led to reduced *G2DHE – EVI1* promoter interaction frequency, indicating that the persistent activity of the oncogenic super-enhancer relies on the constitutive presence and chromatin modulation of PARP1. PARP1-mediated chromatin remodeling has been shown to be a facilitator of binding of the pioneer TF Sox2 to intractable enhancers in closed chromatin regions of embryonic stem cells, making PARP1 a requirement for the expression of pluripotency genes and stemness [37]. Conversely, a recent report indirectly implicated PARP1 in the downregulation of genes involved in immune evasion of leukemic stem cells, since PARPi induced expression of natural killer cell ligands, although the exact mechanism of PARPi-mediated remained obscure [51]. Altogether, these observations confirm the novel molecular function of PARP1 in chromatin regulation, highlight its importance in oncogene regulation in leukemia with enhancer hijacking due to chromosomal rearrangements, and make PARP1 a potential attractive target for further clinical investigation.

## Supplementary information

Supplemental Material

File S1 Supplement ChIP-SICAP results

File S2 Supplement TPM values RNA-Seq

File S3 Supplement differential gene expression
