# Supplementary material for: Identification of therapeutic targets of the hijacked super-enhancer complex in EVI1-rearranged leukemia
Source: Leukemia. 2021 Apr 28;35(11):3127–38. doi: 10.1038/s41375-021-01235-z (PMC8550965; doi:10.1038/s41375-021-01235-z)
Supplement: Supplementary file 1 — Supplemental Material [file 41375_2021_1235_MOESM1_ESM.pdf]

Supplemental methods

Cell culture

MUTZ-3 cells were cultured in 60% MEMalpha (Thermo Fisher Scientific, Waltham, MA, USA), 20% fetal bovine serum (FBS) (Biochrom AG, Berlin, Germany), 20% conditioned medium of 5637 cells, and 10 ng/mL GM-CSF (BIOZOL GmbH, Eching, Germany). UCSD-AML1 cells were cultured in 60% RPMI 1640 (Thermo Fisher Scientific) including the same supplements as MUTZ-3. HNT-34 and MOLM-1 were cultured in 80% RPMI 1640 and 20% FBS. U-937 and K-562 were cultured in 90% RPMI 1640 and 10% FBS. All cultures were supplemented with 1x penicillin/streptomycin (Thermo Fisher Scientific). Metabolic activity was measured by CellTiterGlo assay (Promega, Madison, WI, USA). Cell identity was confirmed by a Multiplex human Cell line Authentication Test and tested for mycoplasma contamination regularly.

Lentiviral transduction

Lentiviral packaging vectors pMD2.G and psPAX2 were a gift from D. Trono (Addgene plasmid #12259 and #12260). Lentiviruses were produced according to standard procedures in Lenti-X 293T cells (Takara Bio, Kusatsu, Japan). For lentiviral transduction, 6-well plates coated with RetroNectin (Takara Bio, Kusatsu, Japan) were used. 500 µL of viral soup was added into a well of a 6-well plate with 2 mL medium and spun down for 45 min at 1 350 g and room temperature (RT). $1x10^6$ cells in 1 mL and 3 µL polybrene (Merck KGaA, Darmstadt, Germany) were added and spun down for 5 min at 314 g and RT. Cells were transduced twice on consecutive days. Puromycin (InvivoGen, San Diego, CA, USA) was added at a concentration of 1 µg/mL two days after the last transduction. Cells were selected for 3 days before samples were harvested.

Western blot

Cells were lysed in lysis buffer (50 mM Tris-HCl pH 7.5, 150 mM NaCl, 1 mM EDTA pH 8.0, 1% (v/v) Triton X-100, 1x complete protease inhibitor (Roche, Basel, Switzerland)) for 20 min

on ice. Debris was pelleted for 30 min at 16 000 g and 4°C, and the supernatant was transferred into a fresh tube. SDS-Page and western blot were carried out according to standard procedures. The following antibodies were used for protein detection: α-EVI1 (#2265; Cell Signaling, Beverly, MA, USA), α-CEBPA D56F10 (#8178; Cell Signaling), α-RUNX1 (sc-365644 X; Santa Cruz Biotechnology, Dallas, TX, USA), α-IKZF1 D10E5 (#9034S; Cell Signaling, Beverly), α-PARP1 (#9542S; Cell Signaling), α-β-Actin (AM1829B; Biomol, Hamburg, Germany), and α-GAPDH (sc-25778; Santa Cruz Biotechnologies). Western blots were imaged on an Odyssey CLx Imaging System (LI-COR Biosciences, Lincoln, NB, USA) using the Image Studio software (LI-COR Biosciences).

Co-Immunoprecipitation

Lysates were prepared as described previously in the western blot section. 75 µg of total protein lysate were used as input control. Per IP, 3 mg of protein was used and diluted with 500 µL IP buffer (1% (v/v) Triton X-100, 0.5% (v/v) NP40, 50 mM Tris-HCl pH8.0, 5 mM EDTA). Triton X-100 was added to a final concentration of 2% (v/v). 10 µL PARP1 antibody (ab227244, Abcam, Cambridge, UK) or 2 µL control IgG (#2729S; Cell Signaling) were added to the lysates and the samples were incubated overnight on a thermoshaker at 4°C and 750 rpm. Per sample, 60 µL Dynabeads Protein G for immunoprecipitation (Thermo Fisher Scientific) were washed with IP buffer and 60 µL beads were added to each IP. Samples were rotated for 3 h at 4°C. Beads were placed on a magnet and washed seven times with wash buffer (20 mM Tris-HCl pH 8.0, 100 mM NaCl, 1 mM EDTA, 0.5% (v/v) NP-40) and two times with LiCl buffer (10 mM Tris-HCl pH 8.0, 1 mM EDTA pH 8.0, 250 mM LiCl, 0.1 % (w/v) Na-deoxycholate, 0.5 % (v/v) NP-40). After washing, the beads were resuspended in 25 µL 1x SDS sample loading buffer with reducing agent and boiled for 10 min at 95°C. Beads were removed from the solution and the eluate was used for western blot analysis.

DNA streptavidin pull-down

Biotinylated DNA probes were generated with PCR using *Taq* DNA polymerase (Qiagen, Venlo, Netherlands) according to the manufacturer's protocol with the primers shown in **Table S1**. The enhancer-containing pGL3 plasmid was used as template for the *G2DHE* probe and MUTZ-3 genomic DNA for the chromosome Y control region. Nuclear lysate of non-fixed MUTZ-3 cells was prepared as described in the ChIP-SICAP methods section using 500 µL lysis buffer (LB) 1, 1 mL LB2, and 100 µL LB3 per 10^7 cells. NaCl was added to a final concentration of 450 mM. The chromatin in the nuclear lysate was sheared mechanically by pulling it at least six times through a 21G and six times through a 23G syringe. Lysates were centrifuged for 30 min at 16 000 g and 4°C. The supernatant was transferred into a new tube and the pellet discarded. 30 µg of the nuclear lysate was used as input control. 1.2 mg of nuclear lysate was used per sample. Each sample was diluted 1:3 with Buffer C (20 mM HEPES-KOH pH 7.9, 1.5 mM $MgCl_2$, 0.2 mM EDTA, 0.5 mM DTT, 1x complete Protease Inhibitor (Roche)) to reach a NaCl concentration of 150 mM. Sonicated salmon sperm DNA was added to the lysate at a final concentration of 100 µg/mL to block unspecific protein-DNA interactions. Samples were rotated for 20 min at 4°C and 25 rpm. 1.2 µg of biotinylated DNA probe was added per sample, and samples were rotated for 30 min at 4°C and 25 rpm. 60 µL streptavidin beads (S1420S; New England Biolabs, Frankfurt am Main, Germany) were added to each sample and samples were rotated for 30 min at RT and 25 rpm. Supernatant was discarded and beads were transferred into a fresh tube with 400 µL Buffer C with 150 mM NaCl. Beads were washed seven times with 400 µL Buffer C with 150 mM NaCl. 30 µL of the last wash step were kept as control. Beads were resuspended in 30 µL 1x NuPAGE LDS Sample Buffer with 1x NuPAGE Sample Reducing Agent (Thermo Fisher Scientific, Waltham, MA, USA) and heated to 95°C for 10 min. Beads were discarded and the supernatant was used for SDS-PAGE and western blot analysis.

Next generation sequencing

ChIP-Seq

Cells were fixed with 1% (v/v) methanol-free formaldehyde (Thermo Fisher Scientific) for 10 min. The reaction was stopped by adding glycine to a final concentration of 125 mM and incubation at RT for 5 min. Nuclear lysates were prepared as described in the ChIP-SICAP section by using 250 µL LB1, 500 µL LB2, and 475 µL LB3 per $5x10^6$ cells. Samples were split in sonication tubes with 150 µL volume per tube. Shearing of the chromatin was performed by sonication in a Bioruptor Pico device (Diagenode, Seraing, Belgium). 220 µL of RIPA buffer I (10 mM Tris-HCl pH 8.0, 1 mM EDTA pH 8.0, 140 mM NaCl, 0.1% (w/v) Na-deoxycholate) were added to each tube. Samples were spun for 10 min at 12 000 g at 4°C. The supernatant was pooled. The pellet was resuspended in 370 µL of RIPA buffer I and spun again. The supernatant was pooled with the previous supernatant. 50 µL of input were taken aside. Dynabeads Protein G for Immunoprecipitation (Thermo Fisher Scientific) were coupled to α-CEBPA D56F10 (#8178; Cell Signaling) antibody and rotated with the sheared chromatin at 4°C overnight. The supernatant was discarded and the beads were transferred into a PCR tube with 180 µL of RIPA buffer with 0.1% (w/v) SDS. The beads were washed five times with RIPA buffer with 0.1% (w/v) SDS, twice with RIPA-500 (10 mM Tris-HCl pH 8.0, 1 mM EDTA pH 8.0, 500 mM NaCl, 1% Triton X-100, 0.1% SDS; 0.1% (w/v) Na-deoxycholate), twice with LiCl wash buffer (10 mM Tris-HCl pH 8.0, 1 mM EDTA pH 8.0, 250 mM LiCl, 0.1% (w/v) Na-deoxycholate, 0.5% NP-40), and twice with 1x TE. The beads were air-dried and eluted in 50 µL direct elution buffer (10 mM Tris-HCl pH 8.0; 10 mM EDTA pH 8.0, 300 mM NaCl, 0.5% (w/v) SDS). Eluates and the input were digested with RNase A (10 mg/ml, Thermo Fisher Scientific), proteinase K (20 mg/mL, Thermo Fisher Scientific), and decrosslinked. Samples were purified with AMPure XP beads (Beckman Coulter, Brea, CA, USA). Library preparation was performed using the NEBNext system for Illumina (New England Biolabs, Frankfurt am Main, Germany) according to the manufacturer's protocol. Samples were sequenced on a HiSeq 2000 v4 platform (Illumina, San Diego, CA, USA) with

50 bp single reads. Reads were aligned to human genome version hg19, and the input was subtracted as background using an in-house pipeline. Integrative Genome Viewer version 2.6.3 (Broad Institute, Cambridge, MA, USA) was used for the visualization. RUNX-1 ChIP-Seq data from *de novo* AML cell patients were published previously and online available (GEO Accession number GSM3734708).[1]

RNA-Seq

Cells were treated with PARP inhibitors or DMSO and harvested at various time points or they were lentivirally transduced with *EVI1* shRNAs and harvested after selection. RNA was extracted using the RNeasy Plus Mini Kit (Qiagen, Venlo, Netherlands), and extracts with RIN values >8 were sent for sequencing. Library preparation was performed according to standard procedures by the DKFZ Genomics and Proteomics Core Facility. Samples were sequenced on an Illumina HiSeq 4000 with paired-end 100 bp reads. Bioinformatic analysis was performed as described below. Published RNA-Seq data from AML samples and cell lines were reanalyzed as described previously.[2,3]

Processing of PARPi and *EVI1* knockdown RNA-Seq data

Reads were aligned to the genome in a 2-pass alignment procedure using STAR aligner (v. 2.7.3a). First, the genome index for the Genome Reference Consortium Human Build version 38 was built with STAR, then reads were aligned, a new index was created using splice junction information from the first pass, and finally, reads were re-aligned to yield the final alignment files in sorted BAM format. Duplicate reads were marked using PICARD MarkDuplicates (v. 2.21.2), and BAM files from multiple sequencing lanes that belong to the same samples were merged using samtools merge (v. 1.9). Gene-level read counting was performed using the Rsubread package (Bioconductor version 3.10). TPM values were then calculated by first normalizing the raw counts per gene length and then scaling them per million.

Determination of differential genes

Top most variable 1 500 genes in the anti-PARP treatment were selected, and clustered into 4 profile clusters using k-means. Differential genes were determined using DESeq2, using the following design: (1) for the anti-PARP treatment experiment, time and treatment type were used as variables, including an interaction term. We selected genes for which the interaction term was significant (2) for the shEVI1 experiments, we considered the time-points (day 3 and day 6) as replicates, and determined the differential genes between shRNA treatment and control.

4C-Seq

4C experiments were conducted as described previously.[2,4] DpnII was used as first cutter and Csp6I as second cutter. The primers shown in **Table S5** were used for library generation. Sequencing was performed on a HiSeq 2000 v4 platform (Illumina) with 50 bp single reads. 4C-Seq fastq files were demultiplexed using the viewpoint specific barcodes. Reads were trimmed using TrimGalore, and aligned using BWA on the hg19 genome. Aligned reads were processed using a custom made pipeline implementing methods inspired by the FourCSeq package in order to produce smoothed bigwig files obtained by smoothing the signal over a number of neighbouring fragments (typically 21).[5] Integrative Genome Viewer version 2.6.3 (Broad Institute) was used for the visualization.

ChIP-SICAP

Cells were fixed with 1.5% (v/v) methanol-free formaldehyde for 15 min, and the reaction was stopped with 125 mM glycine. Instead of permeabilizing the fixed cells with Triton X-100 in TE buffer, a two-step lysis protocol was used. For this, the outer cell membrane was lysed for 10 min in 1 mL of LB1 (50 mM HEPES-KOH pH 7.5, 140 mM NaCl, 1 mM EDTA, 10% (v/v) glycerol, 0.5% (v/v) NP40, 0.25% (v/v) Triton X-100, 1x complete protease inhibitor; Roche) per 25×10^6 cells and then centrifuged for 2 min at 1 000 g and 4°C. The nuclear pellet was incubated with 2 mL LB2 (10 mM Tris-HCl pH 8.0, 200 mM NaCl, 1 mM EDTA, 0.5 mM EGTA, 1x complete protease inhibitor) for 5 min, centrifuged again and the pellet was

resuspended in 600 µL LB3 (10 mM Tris-HCl pH 8.0, 100 mM NaCl, 1 mM EDTA, 0.5 mM EGTA, 0.1% (w/v) Na-deoxycholate, 0.5% (w/v) N-lauroylsarcosine, 1x complete protease inhibitor) to extract the nuclear fraction. Sonication was performed in tubes with 300 µL volume on a Bioruptor Pico (Diagenode, Seraing, Belgium) device with 30 s ON/ 30 s OFF intervals for 20-30 cycles. Triton X-100 was added to a final concentration of 1% (v/v), and 1-5% of total sample volume was taken as input control. The respective amounts of antibodies were added to each sample: 14 µL of α-CEBPA D56F10 (#8178; Cell Signaling, Beverly, MA, USA), 5 µg of α-RUNX1 (ab23980; Abcam, Cambridge, UK), and 5 µg of control IgG (#2729S; Cell Signaling). Samples were vortexed and incubated overnight at 750 rpm and 4°C in a thermomixer. 500 µL IP buffer (50 mM Tris-HCl pH 7.5-8, 5 mM EDTA, 1% (v/v) Triton X-100, 0.5% (v/v) NP40) and 60 µL magnetic Dynabeads Protein G for immunoprecipitation (Thermo Fisher Scientific) were added per sample, and the sample was head-to-tail rotated for 3 h at 4°C. Beads were washed and sheared chromatin was biotinylated, followed by an additional wash step as published previously.[6] Elution of the complexes from the protein G beads was done for 30 min instead of 15 min with 7.5% (w/v) SDS and 200 mM 1,4-dithiothreitol (DTT). The supernatant was diluted with 1 mL IP buffer with 150 mM NaCl. LysC-resistant magnetic streptavidin beads (S1420S; New England Biolabs, Frankfurt am Main, Germany) were generated as described previously and 100 µL beads were added to the supernatant.[7] DNA-bound protein complexes were purified as described before, followed by one washing step with isopropanol and one with acetonitrile wash buffer, respectively.[6] Digestion of the eluted proteins was performed using 300 ng of LysC (12505061; Wako Chemicals, Neuss, Germany) for 16 h at 37°C. Digested peptides and the DNA fraction were cleaned up according to the SP3 protocol as described previously.[6]

Mass spectrometry

Cleaned up peptides were loaded on a trap column (PepMap100 C18 Nano-Trap 100 µm x 2 cm) and separated over a 25 cm analytical column (Waters nanoEase BEH, 75 µm x

250 mm, C18, 1.7 µm, 130 Å,) using the Thermo Easy nLC 1200 nanospray source (Thermo EasynLC 1200; Thermo Fisher Scientific). Solvent A was water with 0.1% formic acid and solvent B was 80% acetonitrile, 0.1% formic acid. During the elution step, the percentage of solvent B increased in a linear fashion from 3% to 8% in 4 min, then increased to 10% in 2 min, to 32% in 68 min, to 50% in 12 min, and finally to 100% in a further 1 min and went down to 3% for the last 11 min. Peptides were analyzed on a Tri-Hybrid Orbitrap Fusion mass spectrometer (Thermo Fisher Scientific) operated in positive (+2 kV) data dependent acquisition mode with HCD fragmentation. The MS1 and MS2 scans were acquired in the Orbitrap and ion trap, respectively with a total cycle time of 3 s. MS1 detection occurred at 120 000 resolution, AGC target 1E6, maximal injection time 50 ms and a scan range of 375-1 500 m/z. Peptides with charge states 2 to 4 were selected for fragmentation with an exclusion duration of 40 s. MS2 occurred with CE 33%, detection in topN mode, and scan rate was set to Rapid. AGC target was 1E4 and maximal injection time allowed of 50 ms. Data were recorded in centroid mode.

Bioinformatic analysis of ChIP-SICAP data

Raw files were analyzed using MaxQuant version 1.5.3.30 (Max-Planck-Institute of Biochemistry, Martinsried, Germany) including the Andromeda search engine.[8,9] Peptides were identified using the Uniprot *Homo sapiens* database concatenated to a database containing protein sequences of contaminants. Default MaxQuant parameters were used with the following modifications: digestion with LysC, default variable modification (methionine oxidation and N-terminal acetylation), cytosine carbamidomethylation as fixed modification, and minimum peptide length 5. FDR was set to 1% at both protein and peptide level. Match between runs option was enabled, Label Free Quantification (LFQ) and iBAQ calculated. Further bioinformatics analyses were performed using an in-house pipeline as follows: known contaminants were removed and fold change of the bait sample intensities over the IgG control intensities was computed. The maximum ratio was limited to 20 and the minimum ratio to 0.5. P-values were calculated with the Limma package and adjusted to

Benjamini-Hochberg (adjusted p-value).[10] Identified proteins with an adj. p-value≤0.10 were considered relevant. Pathway enrichment analysis was performed using DAVID 6.8 (https://david.ncifcrf.gov/home.jsp).[11,12]

Quantitative PCR

The DNA fractions of ChIP-SICAP experiments were purified with AMPure XP beads (Beckman Coulter). ChIP-SICAP qPCR was performed using the TB Green Premix DimerEraser master mix (Takara Bio, Kusatsu, Japan) according to the manufacturer's protocol with the primers shown in **Table S1**. Samples were analyzed with a Light Cycler 480 system (Roche).

For RT-qPCR, mRNA was extracted using the RNeasy Plus Kit (Qiagen, Venlo, Netherlands) and transcribed into cDNA using the SuperScript III First-Strand Synthesis System (Thermo Fisher Scientific) according to the manufacturer's protocols. iTaq Universal SYBR Green Supermix (Bio-Rad Laboratories, Hercules, CA, USA) was used for qPCR according to the manufacturer's protocol with the primers shown in **Table S1**. Samples were run on a CFX96 system (Bio-Rad Laboratories) and analyzed using the CFX Maestro Software (Bio-Rad Laboratories).

May-Grünwald-Giemsa staining

$8x10^4$ cells were resuspended in 150 µL PBS and fixed on a slide using a Shandon CytoSpin 3 device (Thermo Fisher Scientifc) for 5 min at 500 rpm. Slides were stained with May-Grünwald (Sigma-Aldrich, St. Louis, MO, USA) for 3 min, washed three times for 1 min with water, stained with Giemsa (Sigma-Aldrich, St. Louis, MO, USA) for 10 s and washed four times for 1 min with water. The slides were mounted with a drop of Entellan (Merck Millipore, Burlington, MA, USA) and dried for 24 h. Samples were imaged on a Cell Observer (Zeiss, Oberkochen, Germany) with 40x oil objective using the software ZEN blue (Zeiss).

Flow cytometry

Apoptosis staining was carried out according to the FITC Annexin V Apoptosis Detection Kit I (BD Bioscience, San Jose, CA, USA) manufacturer's protocol with the following modifications: 2.5 µL Annexin-V antibody were used, 7-aminoactinomycin (7-AAD) was used instead of propidium iodide.

For staining with differentiation markers, $1x10^6$ cells were resuspended in 50 µL FACS Buffer (PBS with 2% FBS). Antibodies against cell surface markers were added (CD34-PerCPCy5.5, #343611; Biolegend, San Diego, CA, USA), CD11b-APC (#301309; BioLegend), CD14-APCH7 (MϕP9; BD Biosciences), and the samples were incubated for 15 min at 4°C in the dark. 150 µL of 1 µg/mL DAPI (4',6-diamidino-2-phenylindole) solution (BSBTAR1176; VWR, Darmstadt, Germany) was added to 50 µL of cells, and the samples were incubated for 5 min at 4°C in the dark. Cells were washed with 3 mL PBS with 5% FBS and resuspended in 100 µL fixation medium (FIX & PERM Reagent A; Thermo Fisher Scientific). The samples were incubated for 15 min at RT in the dark and washed once with 3 mL PBS with 5% FBS. The cells were resuspended in 100 µL permeabilization medium (FIX & PERM Reagent B, Thermo Fisher Scientific), and the intracellular antibody (cMPO-FITC, sc-51741 FITC; Santa Cruz Biotechnology, Dallas, TX, USA or γH2AX-Alexa488, 560445, BD Biosciences) was added. The samples were incubated for 20 min at RT in the dark and washed with 3 mL PBS with 5% FBS. Cells were resuspended in 300 µL FACS buffer. All samples were acquired on a FACSCelesta device (BD Biosciences) using FACSDiva software (BD Biosciences).

Statistical analysis and data presentation

Unless otherwise stated, statistical significance was calculated using two-sided two-sample t-tests or two-sided one-sample t-tests. Values were assumed to be normally distributed. Exact replicate numbers for the DLA assays are shown in **Table S6**.

Plots were generated using the ggplot2 R package version 3.3.2.[13] Boxplots show the median. The lower and upper hinges of the boxplots correspond to the first and third quartiles

and the whiskers extend to the smallest and largest value as long as their distance from the hinge is smaller than 1.5× inter-quartile range.

Sequence logos were generated using seqLogo R package version 1.56.0.[14] FACS plots were generated with the FACSDiva or FlowJo software. The Integrative Genomics Viewer (IGV) was used for visualization of ChIP-Seq and 4C-Seq tracks. Affinity Designer was used for illustrations.

Supplemental tables

Table S1. List of oligonucleotides and primers with name, sequences, and applied method. PD = pull-down

Name	Sequence	Method
**EnhCore-for**	tttGGATCCACTCCAAGCACCTGCCAAGGC	cloning pGL3
**EnhCore-rev**	tttGTCGACCTCCAGGTGTCCAGAGCCCG	cloning pGL3
**EnhRight-for**	tttGGATCCGAAGGCCTGGGGATGGTGAGG	cloning pGL3
**EnhLeft-rev**	tttGTCGACCCTCACCATCCCCAGGCCTTC	cloning pGL3
**5´mirE-XhoI**	TACAATACTCGAGAAGGTATATTGCTGTTGACAGTGAGCG	miR-E cloning
**3´mirE-EcoRI**	TTAGATGAATTCTAGCCCCTTGAAGTCC GAGGCAGTAGGCA	miR-E cloning
**SGEP-SEQ**	TGTTTGAATGAGGCTTCAGTAC	sequencing
**pGL3-p300-for**	GATGAGTTTGGACAAACCAC	sequencing
**pGL3-p300-rev**	GAGCTGACTGGGTTGAAG	sequencing
**qPCR-Enh-for**	CTCCCTGCATCCGTTACTTG	ChIP-qPCR
**qPCR-Enh-rev**	CAGCCTGCTTTACCACATCA	ChIP-qPCR
**ChIP-CTRL1-for**	TACTAACTTCGTGGTGGCGT	ChIP-qPCR
**ChIP-CTRL1-rev**	TGGAGTGAAAGGCCCTGAAA	ChIP-qPCR
**ChIP-CTRL2-for**	TAGGTAGCGTGCTGTTTGGG	ChIP-qPCR
**ChIP-CTRL2-rev**	TGCTCTCAATGCAACCCTGA	ChIP-qPCR
**qPCR-EVI1-for**	AGTGCCCTGGAGATGAGTTG	RT-qPCR
**qPCR-EVI1-rev**	CTGCTTAAGTTCCTCTGGCAC	RT-qPCR
**qPCR-PBGD-for**	GGCAATGCGGCTGCAA	RT-qPCR
**qPCR-PBGD-rev**	GGGTACCCACGCGAATCAC	RT-qPCR
**PD-Enh-for**	CCACAGGCAGTGGACGG	PD PCR
**PD-Enh-rev**	[Biotin]-ATCCCCAGGCCTTCACATC	PD PCR
**PD-ChrY-For**	TGTAGCCACTTCAAGGACTCT	PD PCR
**PD-ChrY-Rev**	[Biotin]-ACGAGATGTGTCCACCTACT	PD PCR

Table S2. List of mutagenesis primers with mutant number (Mut) and sequences. Mutations are marked in lowercase letters.

Mut	Sequence
**1-for**	GGGGACATGCCACCCCgatgGGCAGTGGACGGGGCC
**1-rev**	GGCCCCGTCCACTGCCcatcGGGGTGGCATGTCCCC
**2-for**	CCCACAGGCAGTGcAtGGGGCCTCCCTGC
**2-rev**	GCAGGGAGGCCCCaTgCACTGCCTGTGGG
**3-for**	CCAGACTCTGCCCACCGCACAGgAGctccAGATAGTTTGTGAAATACCCTG
**3-rev**	CAGGGTATTTCACAAACTATCTggagCTcCTGTGCGGTGGGCAGAGTCTGG
**4-for**	CGCACAGCAGTGATAGATAGTaacTGAcATACCCTGAGCTTGCGGTTGA
**4-rev**	TCAACCGCAAGCTCAGGGTATgTCAgttACTATCTATCACTGCTGTGCG
**5-for**	GATAGATAGTTTGTGAAATACCCTGAGCacGCttTTGACCAGAATTCTGAAAT AATTCCATT
**5-rev**	AATGGAATTATTTCAGAATTCTGGTCAAaaGCgtGCTCAGGGTATTTCACAAA CTATCTATC
**6-for**	CCTGAGCTTGCGGTTGACCAGAAccCTGAgATAATTCCATTGAGACCGGAATTC
**6-rev**	GAATTCCGGTCTCAATGGAATTATgTCAGggTTCTGGTCAACCGCAAGCTCAGG
**7-for**	CCATTGAGACgtacATTCATGCAATGTTCTGTATCCTGATGTGGTAAAGCAGGCTGCCC
**7-rev**	GCATGAATgtacGTCTCAATGGAATTATTTCAGAATTCTGGTCAACCGCAAGCTCAGGGT
**8-for**	CCGGAATTCATGCAATGTTCTGTATtgcGcaGTaGTAAAGCAGGCTGCCCGCGGGGG
**8-rev**	CCCCCGCGGGCAGCCTGCTTTACtACtgCgcaATACAGAACATTGCATGAATTCCGG
**9-for**	GGGGCTTCTGCCACCCtttCTCGCatACGGGCTCCGGAAGGC
**9-rev**	GCCTTCCGGAGCCCGTatGCGAGaaaGGGTGGCAGAAGCCCC
**10-for**	CGCCCACGGGCTCatGAAGGCCCATCTG
**10-rev**	CAGATGGGCCTTCatGAGCCCGTGGGCG
**11-for**	CGGGCTCCGGAAGGCCCATCTagtAGGAGCAGGGC
**11-rev**	GCCCTGCTCCTactAGATGGGCCTTCCGGAGCCCG
**12-for**	TCTGACAGGAGCAGGGCCCTtggccGCACCACCTGACCAGGAG
**12-rev**	CTCCTGGTCAGGTGGTGCggccaAGGGCCCTGCTCCTGTCAGA
**13-for**	GGAGCAGGGCCCTGATAAGggtatgCTGACCAGGAGGCCCCCGG
**13-rev**	CCGGGGGCCTCCTGGTCAGcataccCTTATCAGGGCCCTGCTCC
**14-for**	CCCCCGGCAGAGAGATTAATGCACAAATTacgcACAACCTCCCCATGC
**14-rev**	GCATGGGGAGGTTGTgcgtAATTTGTGCATTAATCTCTCTGCCGGGGG
**15-for**	CCGCAGAGGAGCAGGaCaaGGGGGCACTGGGAAA
**15-rev**	TTTCCCAGTGCCCCCttGtCCTGCTCCTCTGCGG
**16-for**	GCACTGGGAAAGGAAGtttAGAGGAGGCCTCACCC
**16-rev**	GGGTGAGGCCTCCTCTaaaCTTCCTTTCCCAGTGC
**17-for**	GGCCTGGCCTCCCGaccCCTCTCCTAAGCCC
**17-rev**	GGGCTTAGGAGAGGggtCGGGAGGCCAGGCC
**18-for**	GCAGGGGACACGCCATgcaTCTGCAGAGGCAGG
**18-rev**	CCTGCCTCTGCAGAtgcATGGCGTGTCCCCTGC
**19-for**	GCCACCCCCACAGGtgaTGGACGGGGCCTCC
**19-rev**	GGAGGCCCCGTCCAtcaCCTGTGGGGGTGGC
**20-for**	GCCCACCGCACAGCAGTGATAGATccaTTGTGAAATACCCTGA
**20-rev**	TCAGGGTATTTCACAAtggATCTATCACTGCTGTGCGGTGGGC

**21-for**	TTGTGAAATACCCTGAGCTTGCGccgGACCAGAATTCTGAAATAATTCC
**21-rev**	GGAATTATTTCAGAATTCTGGTCcggCGCAAGCTCAGGGTATTTCACAA

Table S3. List of 97-mer oligonucleotides used for miR-E cloning. Sequences were extracted from the Genome-wide Sensor-Based shRNA Prediction by Fellmann *et al*. (see ID).[15] The targeted protein and the sequence are given. SCR = Scrambled control.

shRNA ID	Target	Sequence
**CEBPA.2538**	CEBPA	TGCTGTTGACAGTGAGCGATCACTCTAGATGTTTTATGTATAGTGAAGC<br>CACAGATGTATACATAAAACATCTAGAGTGAGTGCCTACTGCCTCGGA
**CEBPA.1471**	CEBPA	TGCTGTTGACAGTGAGCGATGCCTTGGAAATGCAAACTCATAGTGAAGCC<br>ACAGATGTATGAGTTTGCATTTCCAAGGCACTGCCTACTGCCTCGGA
**RUNX1.524**	RUNX1	TGCTGTTGACAGTGAGCGCTCGGCAGAAACTAGATGATCATAGTGAAGC<br>CACAGATGTATGATCATCTAGTTTCTGCCGATTGCCTACTGCCTCGGA
**RUNX1.468**	RUNX1	TGCTGTTGACAGTGAGCGAACCTACCACAGAGCCATCAAATAGTGAAGC<br>CACAGATGTATTTGATGGCTCTGTGGTAGGTGTGCCTACTGCCTCGGA
**/**	SCR	TGCTGTTGACAGTGAGCGCCACTTCAGATCGTAAGGAGAATAGTGAAGC<br>CACAGATGTATTCTCCTTACGATCTGAAGTGATGCCTACTGCCTCGGA
**Ren.713**	*Renilla* (REN)	Already present in SGEP empty vector

Table S4. Known CEBPA and RUNX1 interactors identified by ChIP-SICAP with references of previous reports.

Protein	Interactor	References
**CEBPA**	CEBPB	Cao *et al*. (1991)[16], Lin *et al*. (2005)[17], Tomaru *et al*. (2009)[18]
	CEBPG	Reinke *et al*. (2013)[19]
	EP300	Rodríguez-Ubreva *et al*. (2012)[20]
	PARP1	Yin *et al*. (2006)[21]
	SMARCD2	Grebien *et al*. (2015)[22]
**RUNX1**	ARID1A	Huber *et al*. (2017)[23]
	CBFB	Kitabayashi *et al*. (2001)[24], Shia *et al*. (2012)[25], Nguygen *et al*. (2005)[26], Philipot *et al*. (2010)[27], Imai *et al*. (2004)[28], Yamaguchi *et al*. (2004)[29], Kim *et al*. (2013)[30], Leong *et al*. (2016)[31], Warren *et al*. (2000)[32], Yonezawa *et al*. (2017)[33], Luck *et al*. (2020)[34], Yu *et al*. (2012)[35]
	DPF2	Huber *et al*. (2017)[23]
	EP300	Yamaguchi *et al*. (2004)[29], Aikawa *et al*. (2006)[36]
	FUBP1	Huber *et al*. (2017)[23]
	HNRNPR	Huber *et al*. (2017)[23]
	HNRNPU	Huber *et al*. (2017)[23]
	IKZF1	Zhou *et al*. (2019)[37]
	KHSRP	Huber *et al*. (2017)[23]
	NONO	Huber *et al*. (2017)[23]
	RBBP4	Yu *et al*. (2012)[35]
	RPA1	Huber *et al*. (2017)[23]
	SET	Yu *et al*. (2012)[35]
	SMARCC1	Huber *et al*. (2017)[23], Bakshi *et al*. (2010)[38], Yu *et al*. (2012)[35]
	SMARCE1	Yu *et al*. (2012)[35]
	TAL1	Hu *et al*. (2009)[39], Yu *et al*. (2012)[35]
	TKT	Huber *et al*. (2017)[23]

Table S5. List of primers used for 4C library preparation. The same reverse primer was used for all samples.

Name	Sequence	sample
**EVI1 prom_R1**	CAAGCAGAAGACGGCATACGAATGCAATGAATTCTCCATTC	all
**EVI1 prom_GC_F6**	AATGATACGGCGACCACCGAACACTCTTTCCCTACACG ACGCTCTTCCGATCTgcATTATTTAGAAGACCTGATC	DMSO
**EVI1 prom_CG_F4**	AATGATACGGCGACCACCGAACACTCTTTCCCTACACG ACGCTCTTCCGATCTcgATTATTTAGAAGACCTGATC	tala
**EVI1 prom_GT_F3**	AATGATACGGCGACCACCGAACACTCTTTCCCTACACG ACGCTCTTCCGATCTgtATTATTTAGAAGACCTGATC	ola

Table S6. List of exact number of biological replicates (n) for each DLA condition.

Experiment	Figure	Condition	n
**DLA *G2DHE* fragments**	1b	all	4
**DLA *G2DHE* mutants**	1c	pGL3, Prom, wildtype *G2DHE*	13
		Mut 1, 5	5
		Mut 2, 7, 9, 10, 11, 12, 15, 17, 19, 20, 21	3
		Mut 3, 8	4
		Mut 4, 6	8
		Mut 13	9
		Mut 14, 16, 18	6
	S1b	HNT-34:	
		pGL3, Prom, wildtype *G2DHE*	13
		Mut 1, 5	5
		Mut 4, 6	8
		Mut 1+5, 4+6	3
		Mut 8	4
		MUTZ-3:	
		All conditions	5
		MOLM-1:	
		pGL3, Prom, wildtype G2DHE, Mut 1, 4, 5, 6, 8	6
		Mut 1+5, 4+6	5

Supplemental references

1 Gerritsen M, Yi G, Tijchon E, Kuster J, Schuringa JJ, Martens JHA *et al.* RUNX1 mutations enhance self-renewal and block granulocytic differentiation in human in vitro models and primary AMLs. *Blood Adv* 2019; **3**: 320–332.

2 Gröschel S, Sanders MA, Hoogenboezem R, De Wit E, Bouwman BAM, Erpelinck C *et al.* A single oncogenic enhancer rearrangement causes concomitant EVI1 and GATA2 deregulation in Leukemia. *Cell* 2014; **157**: 369–381.

3 Gröschel S, Sanders MA, Hoogenboezem R, Zeilemaker A, Havermans M, Erpelinck C *et al.* Mutational spectrum of myeloid malignancies with inv(3)/t(3;3) reveals a predominant involvement of RAS/RTK signaling pathways. *Blood* 2015; **125**: 133–139.

4 van de Werken, HJ, Landan G, Holwerda SJ, Hoichman M, Klous P, Chachik R, Splinter E *et al.* Robust 4C-seq data analysis to screen for regulatory DNA interactions. *Nat Methods* 2012; **10**: 969–972.

5 Klein FA, Pakozdi T, Anders S, Ghavi-Helm Y, Furlong EEM, Huber W. FourCSeq: analysis of 4C sequencing data. *Bioinformatics* 2015; **31**: 3085–3091.

6 Rafiee MR, Girardot C, Sigismondo G, Krijgsveld J. Expanding the Circuitry of Pluripotency by Selective Isolation of Chromatin-Associated Proteins. *Mol Cell* 2016; **64**: 624–635.

7 Rafiee M, Sigismondo G, Kalxdorf M, Förster L, Brügger B, Béthune J *et al.* Protease-resistant streptavidin for interaction proteomics. *Mol Syst Biol* 2020; **16**. doi:10.15252/msb.20199370.

8 Cox J, Mann M. MaxQuant enables high peptide identification rates, individualized p.p.b.-range mass accuracies and proteome-wide protein quantification. *Nat Biotechnol* 2008; **26**: 1367–1372.

9 Tyanova S, Temu T, Cox J. The MaxQuant computational platform for mass spectrometry-based shotgun proteomics. *Nat Protoc* 2016; **11**: 2301–2319.

10 Matthew E Ritchie, Belinda Phipson, Di Wu, Yifang Hu, Charity W Law, Wei Shi *et al.* Limma Powers Differential Expression Analyses for RNA-sequencing and Microarray Studies - PubMed. *Nucleic Acids Res* 2015; **43**: e47.

11 Huang DW, Sherman BT, Lempicki RA. Bioinformatics enrichment tools: Paths toward the comprehensive functional analysis of large gene lists. *Nucleic Acids Res* 2009; **37**: 1–13.

12 Huang DW, Sherman BT, Lempicki RA. Systematic and integrative analysis of large gene lists using DAVID bioinformatics resources. *Nat Protoc* 2009; **4**: 44–57.

13 Wickham H. *ggplot2: Elegant Graphics for Data Analysis.* Springer-Verlag New York., 2016https://ggplot2.tidyverse.org (accessed 15 Dec2020).

14 Bembom O, Ivanek R. seqLogo: Sequence logos for DNA sequence alignments. 2020.https://bioconductor.org/packages/release/bioc/html/seqLogo.html (accessed 15 Dec2020).

15 Fellmann C, Hoffmann T, Sridhar V, Hopfgartner B, Muhar M, Roth M *et al.* An optimized microRNA backbone for effective single-copy RNAi. *Cell Rep* 2013; **5**: 1704–1713.

16 Cao Z, Umek RM, McKnight SL. Regulated expression of three C/EBP isoforms during adipose conversion of 3T3-L1 cells. *Genes Dev* 1991; **5**: 1538–1552.

17 Lin L, Qian Y, Shi X, Chen Y. Induction of a cell stress response gene RTP801 by DNA damaging agent methyl methanesulfonate through CCAAT/Enhancer binding protein. *Biochemistry* 2005; **44**: 3909–3914.

18 Tomaru T, Steger DJ, Lefterova MI, Schupp M, Lazar MA. Adipocyte-specific

expression of murine resistin is mediated by synergism between peroxisome proliferator-activated receptor γ and CCAAT/enhancer-binding proteins. *J Biol Chem* 2009; **284**: 6116–6125.

19 Reinke AW, Baek J, Ashenberg O, Keating AE. Networks of bZIP protein-protein interactions diversified over a billion years of evolution. *Science (80-)* 2013; **340**: 730–734.

20 Rodríguez-Ubreva J, Ciudad L, Gómez-Cabrero D, Parra M, Bussmann LH, Di Tullio A *et al.* Pre-B cell to macrophage transdifferentiation without significant promoter DNA methylation changes. *Nucleic Acids Res* 2012; **40**: 1954–1968.

21 Yin H, Glass J. In prostate cancer cells the interaction of C/EBPalpha with Ku70, Ku80, and poly(ADP-ribose) polymerase-1 increases sensitivity to DNA damage. | BioGRID. *J Biol Chem* 2006; **281**: 11496–11505.

22 Grebien F, Vedadi M, Getlik M, Giambruno R, Grover A, Avellino R *et al.* Pharmacological targeting of the Wdr5-MLL interaction in C/EBPα N-terminal leukemia. *Nat Chem Biol* 2015; **11**: 571–578.

23 Huber FM, Greenblatt SM, Davenport AM, Martinez C, Xu Y, Vu LP *et al.* Histone-binding of DPF2 mediates its repressive role in myeloid differentiation. *Proc Natl Acad Sci U S A* 2017; **114**: 6016–6021.

24 Kitabayashi I, Aikawa Y, Nguyen L, Yokoyama A, Ohki M. Activation of AML1-mediated transcription by MOZ and inhibition by the MOZ-CBP fusion protein. | BioGRID. *EMBO J* 2001; **20**: 7184–7196.

25 Shia WJ, Okumura AJ, Yan M, Sarkeshik A, Lo MC, Matsuura S *et al.* PRMT1 interacts with AML1-ETO to promote its transcriptional activation and progenitor cell proliferative potential. *Blood* 2012; **119**: 4953–4962.

26 Nguyen LA, Pandolfi PP, Aikawa Y, Tagata Y, Ohki M, Kitabayashi I. Physical and

functional link of the leukemia-associated factors AML1 and PML. *Blood* 2005; **105**: 292–300.

27 Philipot O, Joliot V, Ait-Mohamed O, Pellentz C, Robin P, Fritsch L *et al.* The core binding factor CBF negatively regulates skeletal muscle terminal differentiation. *PLoS One* 2010; **5**: e9425.

28 Imai Y, Kurokawa M, Yamaguchi Y, Izutsu K, Nitta E, Mitani K *et al.* The Corepressor mSin3A Regulates Phosphorylation-Induced Activation, Intranuclear Location, and Stability of AML1. *Mol Cell Biol* 2004; **24**: 1033–1043.

29 Yamaguchi Y, Kurokawa M, Imai Y, Izutsu K, Asai T, Ichikawa M *et al.* AML1 Is Functionally Regulated through p300-mediated Acetylation on Specific Lysine Residues. *J Biol Chem* 2004; **279**: 15630–15638.

30 Kim DY, Kwon E, Hartley PD, Crosby DC, Mann S, Krogan NJ *et al.* CBFβ Stabilizes HIV Vif to Counteract APOBEC3 at the Expense of RUNX1 Target Gene Expression. *Mol Cell* 2013; **49**: 632–644.

31 Leong WY, Guo H, Ma O, Huang H, Cantor AB, Friedman AD. Runx1 phosphorylation by Src increases trans-activation via augmented stability, reduced histone deacetylase (HDAC) binding, and increased DNA affinity, and activated Runx1 favors granulopoiesis. *J Biol Chem* 2016; **291**: 826–836.

32 Warren AJ, Bravo J, Williams RL, Rabbitts TH. Structural basis for the heterodimeric interaction between the acute leukaemia-associated transcription factors AML1 and CBFβ. *EMBO J* 2000; **19**: 3004–3015.

33 Yonezawa T, Takahashi H, Shikata S, Liu X, Tamura M, Asada S *et al.* The ubiquitin ligase STUB1 regulates stability and activity of RUNX1 and RUNX1–RUNX1T1. *J Biol Chem* 2017; **292**: 12528–12541.

34 Luck K, Kim DK, Lambourne L, Spirohn K, Begg BE, Bian W *et al.* A reference map of

the human binary protein interactome. *Nature* 2020; **580**: 402–408.

35 Yu M, Mazor T, Huang H, Huang HT, Kathrein KL, Woo AJ *et al.* Direct Recruitment of Polycomb Repressive Complex 1 to Chromatin by Core Binding Transcription Factors. *Mol Cell* 2012; **45**: 330–343.

36 Aikawa Y, Nguyen LA, Isono K, Takakura N, Tagata Y, Schmitz ML *et al.* Roles of HIPK1 and HIPK2 in AML1- and p300-dependent transcription, hematopoiesis and blood vessel formation. *EMBO J* 2006; **25**: 3955–3965.

37 Zhou N, Gutierrez-Uzquiza A, Zheng XY, Chang R, Vogl DT, Garfall AL *et al.* RUNX proteins desensitize multiple myeloma to lenalidomide via protecting IKZFs from degradation. *Leukemia* 2019; **33**: 2006–2021.

38 Bakshi R, Hassan MQ, Pratap J, Lian JB, Montecino MA, Van Wijnen AJ *et al.* The human SWI/SNF complex associates with RUNX1 to control transcription of hematopoietic target genes. *J Cell Physiol* 2010; **225**: 569–576.

39 Hu X, Li X, Valverde K, Fu X, Noguchi C, Qiu Y *et al.* LSD1-mediated epigenetic modification is required for TAL1 function and hematopoiesis. *Proc Natl Acad Sci U S A* 2009; **106**: 10141–10146.

40 Garber M, Guttman M, Clamp M, Zody MC, Friedman N, Xie X. Identifying Novel Constrained Elements by Exploiting Biased Substitution Patterns. *Bioinformatics* 2009; **25**: i54-62.

Supplemental figures

Fig. S1. *G2DHE* mutants used for dual luciferase assay. a *In silico* analysis of the *G2DHE* element revealed several putative TF binding sites (TFBS) as identified with the JASPAR2016 and the Alggen Promo (TRANSFAC) tool. The names of the TFs are indicated in bold and the respective database identifier is written in parentheses. Position weight matrices of the human proteins were used for the analysis except for IKZF1 where the murine (m) matrix was used for prediction. Sequence logos are shown for those sites identified with the JASPAR2016 database. The wildtype (WT) and mutant (M) enhancer sequences are shown for each predicted site. For the JASPAR2016 analysis a relative similarity score ≥ 0.80 was considered relevant and for the TRANSFAC database a relative dissimilarity score of < 15% was used. **b** Luciferase reporter assay in three different leukemic cell lines with 3q-rearrangements for chosen mutations (Mut) and their respective TFBS (n≥3). TFBS in parentheses are in close proximity to the mutations without being affected by them. Cells were co-transfected with the luciferase reporter plasmid pGL3 and the *Renilla* control plasmid. pGL3 empty vector (pGL3), pGL3 vector containing the *EVI1* promoter (Prom), and pGL3 vector containing the *EVI1* promoter and the wildtype *G2DHE* were used as controls (CTRL). Luciferase signal was normalized to *Renilla* signal. The relative luciferase signal was further normalized to the signal of the wildtype *G2DHE* (red line). Statistical significance was calculated with two-sided one-sample t-tests.

Fig. S2. CEBPA and RUNX1 expression, association with *G2DHE*, and ChIP-SICAP results. a Western blot to show the expression levels of CEBPA and RUNX1 in different 3q-rearranged and non-3q-rearranged cell lines. **b** Quantification of CEBPA and RUNX1 protein expression of the cell lines shown in a. **c** ChIP-Seq binding profiles of CEBPA and RUNX1 in leukemic cell lines. Light blue track: CEBPA ChIP-Seq peaks in 3q-rearranged MOLM-1. Dark blue track: RUNX1 ChIP-Seq peaks of *de novo* AML cell patient samples (dataset GSM3734708 by Gerritsen *et al.*).[1] Numbers show the data range. Dark grey: Conservation

(SiPhy rate 10 mer).[40] The core enhancer sequence, the qPCR product used for detection in ChIP-SICAP-qPCR, and the pull-down (PD) probe are annotated in black, the predicted CEBPA TFBS in light blue, and the predicted RUNX1 TFBS in dark blue. **d-f** ChIP-SICAP was performed in three different 3q-rearranged cell lines using antibodies against CEBPA and RUNX1, or an unspecific IgG control. Experiments were carried out in duplicates (n=2). **d** The DNA fraction of the ChIP-SICAP experiment was used for enrichment quantification of the *G2DHE* region by qPCR compared to two unrelated control regions (CTRL). **e-f** The protein fraction of the ChIP-SICAP experiments was analyzed by mass spectrometry to identify chromatin-bound interactors of RUNX1 and CEBPA. **e** The fold change of the intensity of CEBPA or RUNX1 samples over the IgG control was calculated for both replicates (R1 and R2). A cut-off of 20 was used for all proteins with a fold change >20. The graph shows the correlation between replicate 1 and 2. Marked in red is the threshold of fold change = 2. **f** Proteins enriched in the CEBPA- or RUNX1-captured samples over the IgG controls were ranked according to their iBAQ intensity.

Fig. S3. GOTERM and BioGRID analysis of proteins identified with ChIP-SICAP. Proteins identified in at least two cell lines per bait protein were fed into the DAVID online tool and analyzed according to their cellular component (CC), biological pathway (BP), and molecular function (MF) according to GOTERM. Known human and murine interactors of CEBPA and RUNX1 were extracted from BioGRID and annotated accordingly. **a** Analysis of CEBPA interactors. **b** Analysis of RUNX1 interactors. **c** Potential true positive (PTP) and potential false positive (PFP) hits. Relative protein intensities (iBAQ) are shown for the average of RUNX1 and CEBPA ChIP-SICAP assays. Proteins were categorized into PTP if they are interactors of CEBPA and RUNX1, chromatin or DNA-binders or if they have nuclear localization. Proteins were categorized as PFP if they are ribosomal proteins or cytoplasmic proteins. Please note that the categories are exclusive meaning that if a protein falls into a category, it is not considered in the next categories.

Fig. S4. Western blot quantification of MUTZ-3 treated with PARPi or *PARP1* shRNAs shows reduction in EVI1 protein levels. a MUTZ-3 cells were treated with 10 µM olaparib (ola), 1 µM talazoparib (tala), or DMSO or were left untreated for 24 h (n=3). Proteins were extracted from cells treated with PARPi and EVI1 was measured by western blot. β-Actin was used as loading control. Protein bands were quantified and normalized to the loading control and DMSO. Statistical significance was calculated using two-sided two-sample t-tests. **b** MUTZ-3 cells were lentivirally transduced with constructs encoding shRNAs against *PARP1* or *Renilla* (REN) and selected with puromycin. Samples were harvested on day 3 of puromycin selection, and protein levels were analyzed by western blot using antibodies against PARP1 and EVI1. β-Actin served as a loading control. A miR-E construct targeting Renilla (REN) and untreated parental cells served as non-targeting controls.

Fig. S5. PARPi sensitivity in different AML cell lines. Cells were treated with the indicated amounts of olaparib or talazoparib (n=3). Metabolic activity was measured as an indicator of cell viability by CellTiter-Glo assay. The values were normalized to those of the 0 h time point and to the DMSO control of each time point.

Fig. S6. RNA-Seq following PARPi in inv(3) AML cells. MUTZ-3 cells were treated with 10 µM olaparib, 1 µM talazoparib, or DMSO, and RNA was harvested for sequencing after 12 h, 24 h, and 48 h. **a** Heatmap of clusters identified. The top most variable 1 500 genes in the samples treated with PARPi were selected, and clustered into 4 profile clusters using k-means. Experiment, time, and treatment type were used as variables. **b-e** Pathway enrichment analysis of the clusters identified in **a**. Shown is the number of genes of the cluster assigned to the pathway (gene count), the proportion of genes from this cluster that were assigned to the pathway (GeneRatio), and the adjusted p-value.

Figure S7. Genes deregulated by PARPi and *EVI1* knockdown as identified by RNA-Seq. a and **c** Commonly downregulated (a) and upregulated (c) genes by PARPi and *EVI1* knockdown as identified by RNA-Seq. Genes included in cluster 3 of the PARPi data were compared to the genes deregulated by *EVI1* knockdown to determine the genes downregulated by both conditions. The same was performed using cluster 4 of the PARPi data for determining upregulated genes. Fold change of gene expression under treatment conditions (PARPi or shEVI1, respectively) over the control (DMSO or non-targeting control, respectively) is shown. **b** and **d** Pathway enrichment analysis of the commonly downregulated (b) and upregulated (d) genes identified in **a** and **c**. Shown is the number of genes assigned to the pathway (gene count), the proportion of genes from this cluster that were assigned to the pathway (GeneRatio), and the adjusted p-value.

Figure S1

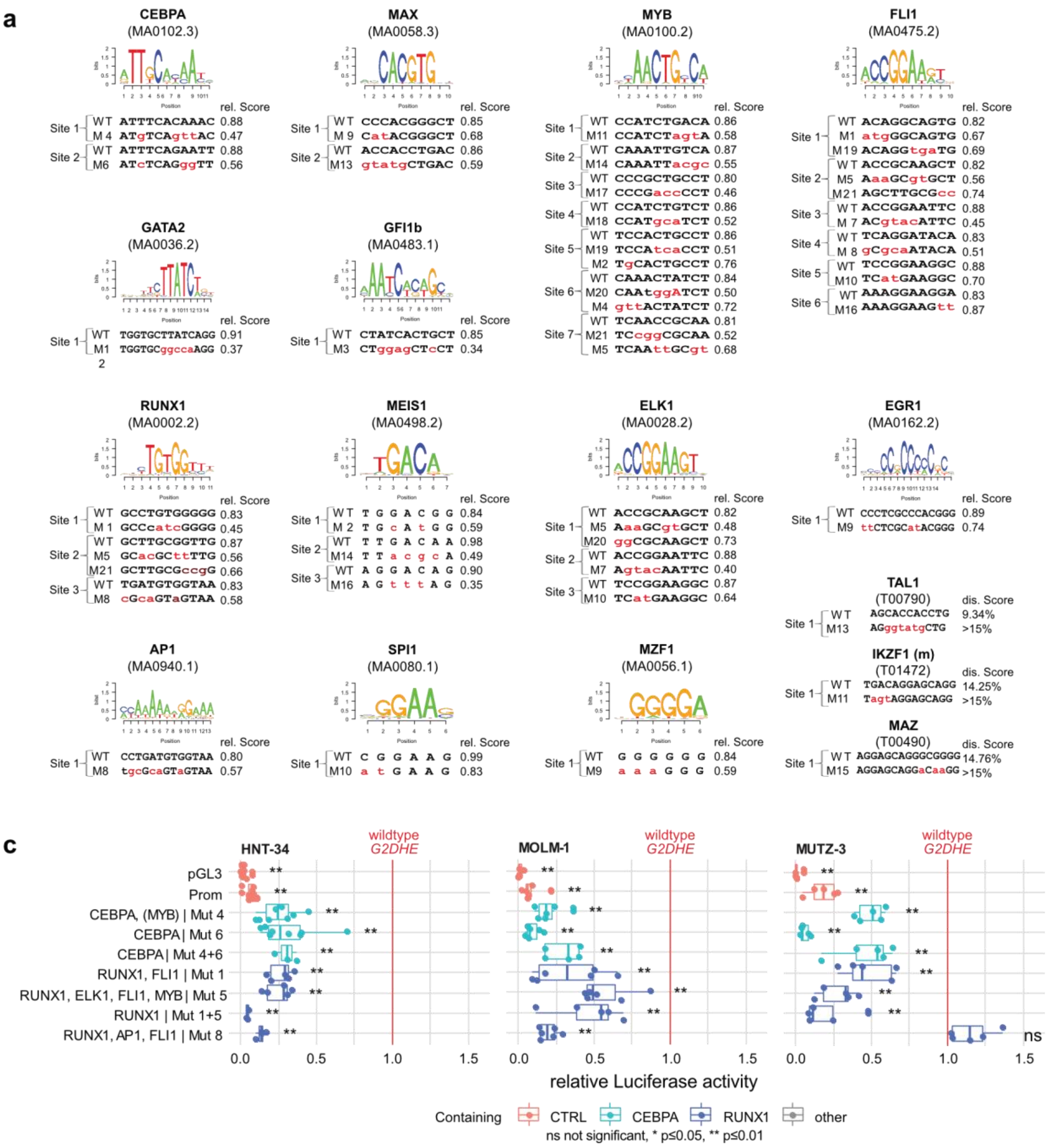

a
CEBPA
(MA0102.3)
rel. Score
Site 1 WT ATTTCACAAAC 0.88
M4 ATgTCAgttAC 0.47
Site 2 WT ATTTCAGAATT 0.88
M6 ATcTCAGggTT 0.56
MAX
(MA0058.3)
rel. Score
Site 1 WT CCCACGGGCT 0.85
M9 CatACGGGCT 0.68
Site 2 WT ACCACCTGAC 0.86
M13 gtatgCTGAC 0.59
MYB
(MA0100.2)
rel. Score
Site 1 WT CCATCTGACA 0.86
M11 CCATCTagtA 0.58
Site 2 WT CAAATTGTCA 0.87
M14 CAAATTacgc 0.55
Site 3 WT CCCGCTGCCT 0.80
M17 CCCGacccCCT 0.46
Site 4 WT CCATCTGTCT 0.86
M18 CCATgcaTCT 0.52
Site 5 WT TCCACTGCCT 0.86
M19 TCCAtcaCCT 0.51
M2 TgCACTGCCT 0.76
Site 6 WT CAAACTATCT 0.84
M20 CAAtggATCT 0.50
M4 gttACTATCT 0.72
Site 7 WT TCAACCGCAA 0.81
M21 TCcggCGCAA 0.52
M5 TCAAttGCgt 0.68
FLI1
(MA0475.2)
rel. Score
Site 1 WT ACAGGCAGTG 0.82
M1 atgGGCAGTG 0.67
M19 ACAGGtgaTG 0.69
Site 2 WT ACCGCAAGCT 0.82
M5 AaaGCgtGCT 0.56
M21 AGCTTGCGcc 0.74
Site 3 WT ACCGGAATTC 0.88
M7 ACgtacATTC 0.45
Site 4 WT TCAGGATACA 0.83
M8 gCgcaATACA 0.51
Site 5 WT TCCGGAAGGC 0.88
M10 TCatGAAGGC 0.70
Site 6 WT AAAGGAAGGA 0.83
M16 AAAGGAAGtt 0.87
GATA2
(MA0036.2)
rel. Score
Site 1 WT TGGTGCTTATCAGG 0.91
M12 TGGTGCggccaAGG 0.37
GFI1b
(MA0483.1)
rel. Score
Site 1 WT CTATCACTGCT 0.85
M3 CTggagCTcCT 0.34
RUNX1
(MA0002.2)
rel. Score
Site 1 WT GCCTGTGGGGG 0.83
M1 GCCcatcGGGG 0.45
Site 2 WT GCTTGCGGTTG 0.87
M5 GCacGCtttTG 0.56
M21 GCTTGCGccgG 0.66
Site 3 WT TGATGTGGTAA 0.83
M8 cGcaGTaGTAA 0.58
MEIS1
(MA0498.2)
rel. Score
Site 1 WT TGGACGG 0.84
M2 TGcAtGG 0.59
Site 2 WT TTGACAA 0.98
M14 TTacgcA 0.49
Site 3 WT AGGACAG 0.90
M16 AGtttAG 0.35
ELK1
(MA0028.2)
rel. Score
Site 1 WT ACCGCAAGCT 0.82
M5 AaaGCgtGCT 0.48
M20 ggCGCAAGCT 0.73
Site 2 WT ACCGGAATTC 0.88
M7 AgtacAATTC 0.40
Site 3 WT TCCGGAAGGC 0.87
M10 TCatGAAGGC 0.64
EGR1
(MA0162.2)
rel. Score
Site 1 WT CCCTCGCCCACGGG 0.89
M9 ttCTCGCatACGGG 0.74
TAL1
(T00790)
dis. Score
Site 1 WT AGCACCACCTG 9.34%
M13 AGggtatgCTG >15%
IKZF1 (m)
(T01472)
dis. Score
Site 1 WT TGACAGGAGCAGG 14.25%
M11 TagtAGGAGCAGG >15%
MAZ
(T00490)
dis. Score
Site 1 WT AGGAGCAGGGCGGGG 14.76%
M15 AGGAGCAGGaCaaGG >15%
AP1
(MA0940.1)
rel. Score
Site 1 WT CCTGATGTGGTAA 0.80
M8 tgcGcaGTaGTAA 0.57
SPI1
(MA0080.1)
rel. Score
Site 1 WT CGGAAG 0.99
M10 atGAAG 0.83
MZF1
(MA0056.1)
rel. Score
Site 1 WT GGGGGG 0.84
M9 aaaGGG 0.59
c
HNT-34
MOLM-1
MUTZ-3
wildtype G2DHE
pGL3
Prom
CEBPA, (MYB) | Mut 4
CEBPA | Mut 6
CEBPA | Mut 4+6
RUNX1, FLI1 | Mut 1
RUNX1, ELK1, FLI1, MYB | Mut 5
RUNX1 | Mut 1+5
RUNX1, AP1, FLI1 | Mut 8
**
ns
0.0
0.5
1.0
1.5
relative Luciferase activity
Containing CTRL CEBPA RUNX1 other
ns not significant, * p≤0.05, ** p≤0.01

505 **Figure S2**

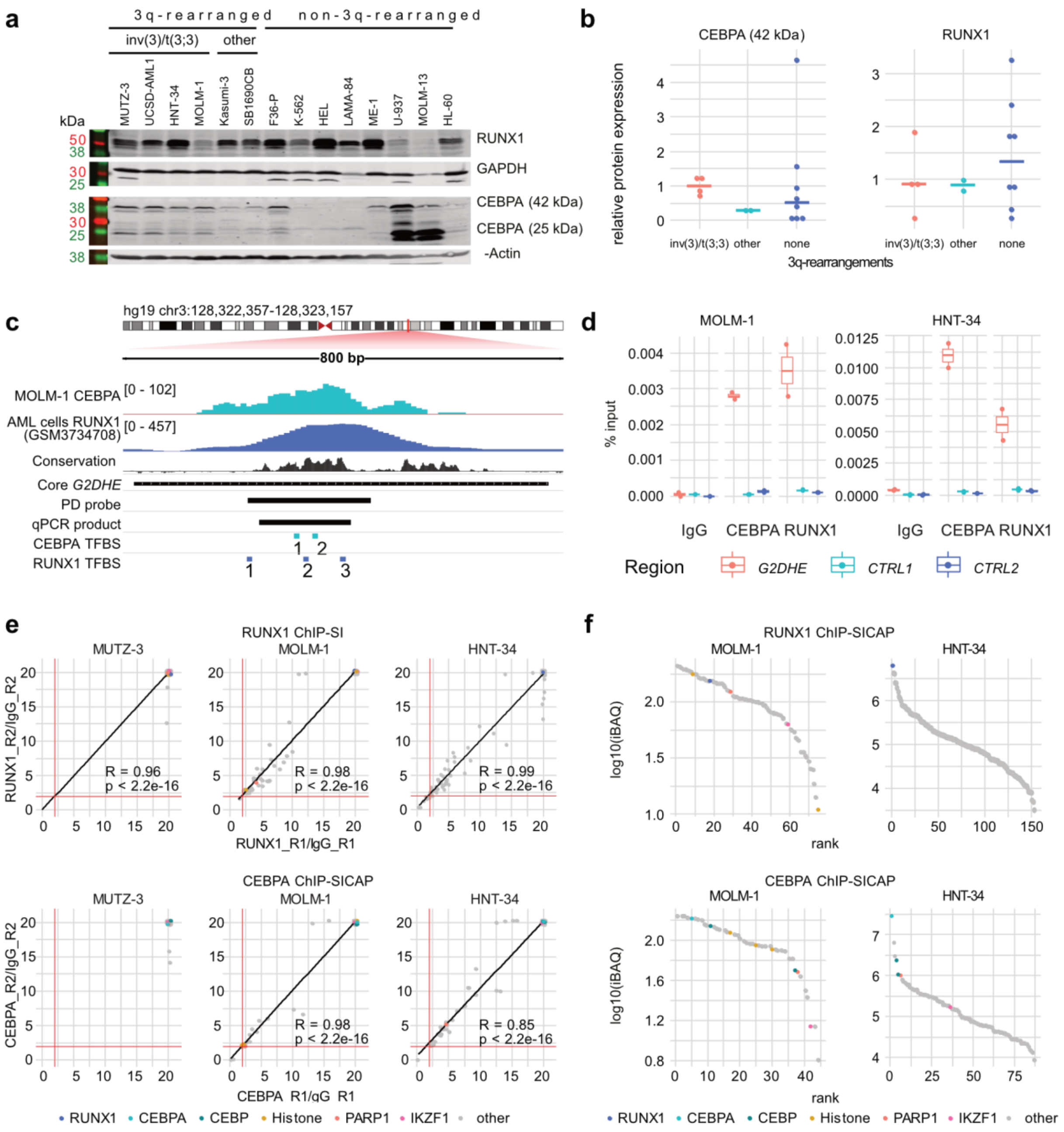

506

Figure S3

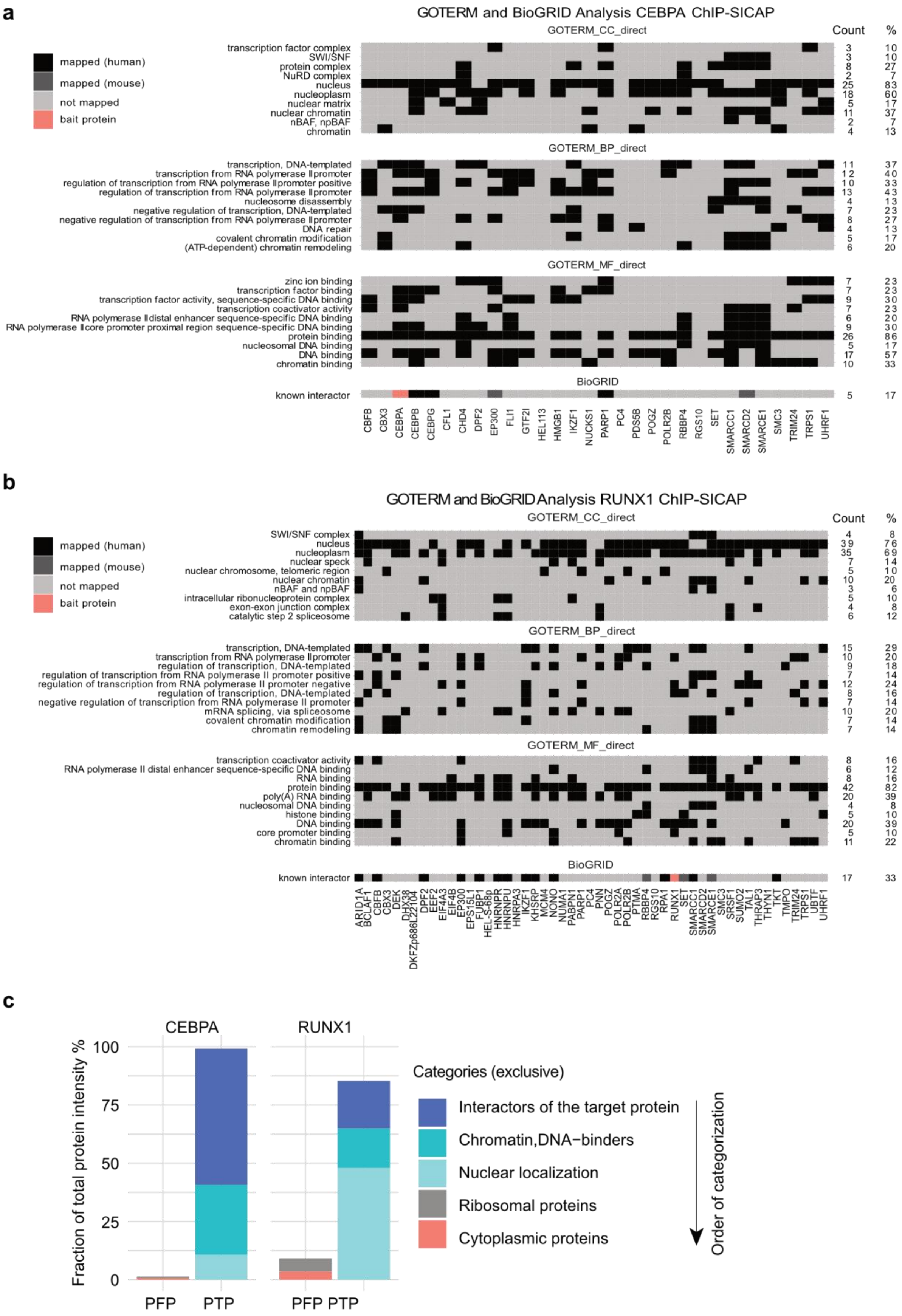

a
GOTERM and BioGRID Analysis CEBPA ChIP-SICAP
mapped (human)
mapped (mouse)
not mapped
bait protein
GOTERM_CC_direct
Count
%
transcription factor complex
SWI/SNF
protein complex
NuRD complex
nucleus
nucleoplasm
nuclear matrix
nuclear chromatin
nBAF, npBAF
chromatin
GOTERM_BP_direct
transcription, DNA-templated
transcription from RNA polymerase II promoter
regulation of transcription from RNA polymerase II promoter positive
regulation of transcription from RNA polymerase II promoter
nucleosome disassembly
negative regulation of transcription, DNA-templated
negative regulation of transcription from RNA polymerase II promoter
DNA repair
covalent chromatin modification
(ATP-dependent) chromatin remodeling
GOTERM_MF_direct
zinc ion binding
transcription factor binding
transcription factor activity, sequence-specific DNA binding
transcription coactivator activity
RNA polymerase II distal enhancer sequence-specific DNA binding
RNA polymerase II core promoter proximal region sequence-specific DNA binding
protein binding
nucleosomal DNA binding
DNA binding
chromatin binding
BioGRID
known interactor
CBFB CBX3 CEBPA CEBPB CEBPG CFL1 CHD4 DPF2 EP300 FLI1 GTF2I HEL113 HMGB1 IKZF1 NUCKS1 PARP1 PC4 PDS5B POGZ POLR2B RBBP4 RGS10 SET SMARCC1 SMARCD2 SMARCE1 SMC3 TRIM24 TRPS1 UHRF1
b
GOTERM and BioGRID Analysis RUNX1 ChIP-SICAP
mapped (human)
mapped (mouse)
not mapped
bait protein
GOTERM_CC_direct
Count
%
SWI/SNF complex
nucleus
nucleoplasm
nuclear speck
nuclear chromosome, telomeric region
nuclear chromatin
nBAF and npBAF
intracellular ribonucleoprotein complex
exon-exon junction complex
catalytic step 2 spliceosome
GOTERM_BP_direct
transcription, DNA-templated
transcription from RNA polymerase II promoter
regulation of transcription, DNA-templated
regulation of transcription from RNA polymerase II promoter positive
regulation of transcription from RNA polymerase II promoter negative
regulation of transcription, DNA-templated
negative regulation of transcription from RNA polymerase II promoter
mRNA splicing, via spliceosome
covalent chromatin modification
chromatin remodeling
GOTERM_MF_direct
transcription coactivator activity
RNA polymerase II distal enhancer sequence-specific DNA binding
RNA binding
protein binding
poly(A) RNA binding
nucleosomal DNA binding
histone binding
DNA binding
core promoter binding
chromatin binding
BioGRID
known interactor
ARID1A BCLAF1 CBFB CBX3 DEK DHX38 DKFZp686L22104 DPF2 EEF2 EIF4A3 EIF4B EP300 EPS15L1 FUBP1 HEL-S-68p HNRNPR HNRNPU HNRPA3 IKZF1 KHSRP MCM4 NONO NUMA1 PABPN1 PARP1 PC4 PNN POGZ POLR2A POLR2B PTMA RBBP4 RGS10 RPA1 RUNX1 SET SMARCC1 SMARCD2 SMARCE1 SMC3 SRSF1 SUMO2 TAL1 THRAP3 THYN1 TKT TMPO TRIM24 TRPS1 UBTF UHRF1
c
CEBPA
RUNX1
Fraction of total protein intensity %
100
75
50
25
0
PFP
PTP
PFP PTP
Categories (exclusive)
Interactors of the target protein
Chromatin,DNA−binders
Nuclear localization
Ribosomal proteins
Cytoplasmic proteins
Order of categorization

Figure S4

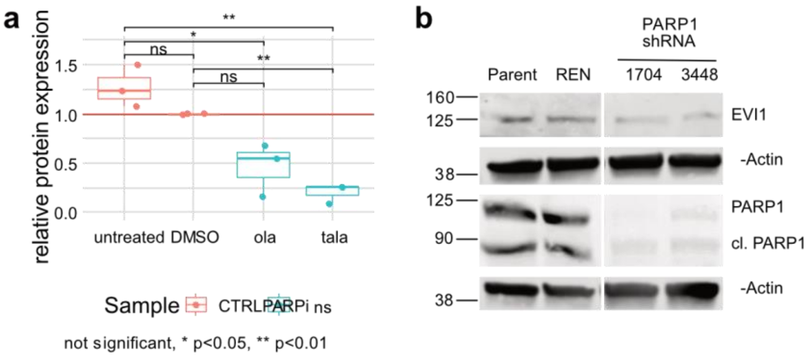

Figure S5

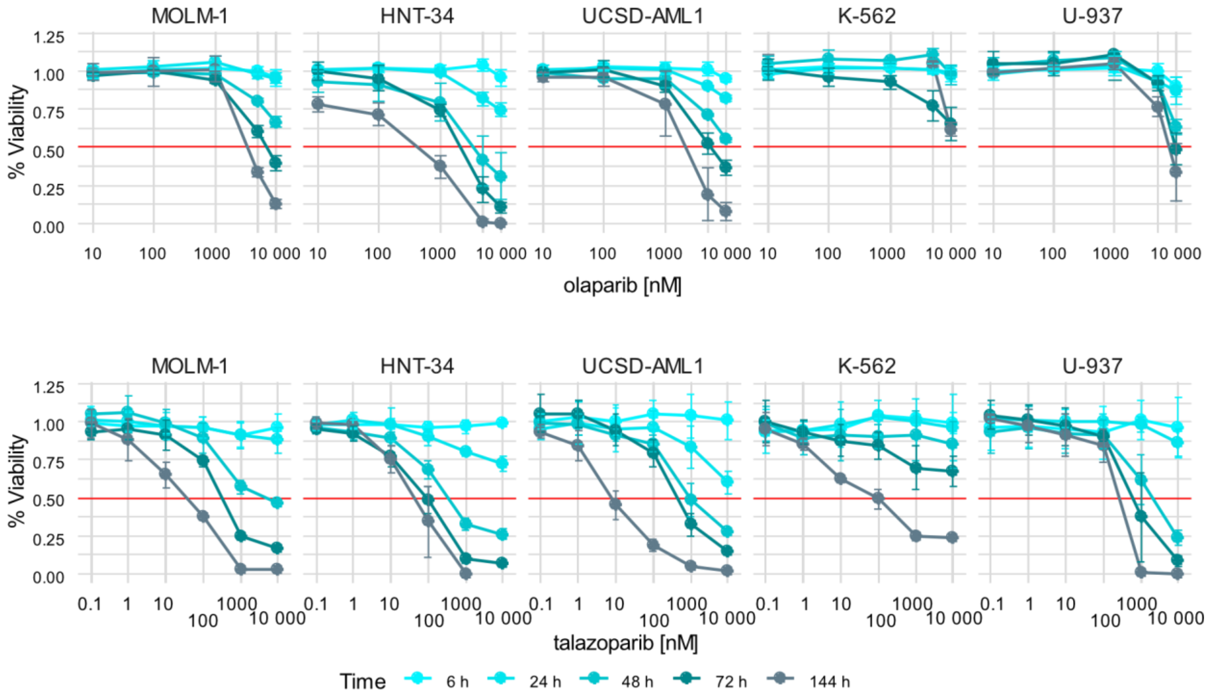

Figure S6

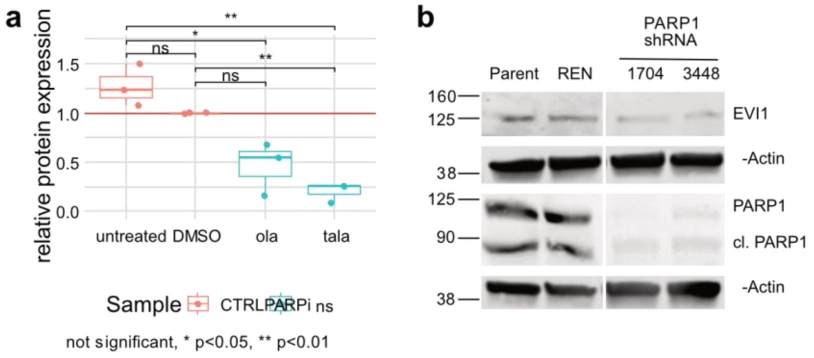
a
relative protein expression
1.5
1.0
0.5
0.0
untreated DMSO ola tala
ns
*
**
ns
**
Sample CTRL PARPi ns
not significant, * p<0.05, ** p<0.01
b
PARP1
shRNA
Parent REN 1704 3448
160
125
38
125
90
38
EVI1
-Actin
PARP1
cl. PARP1
-Actin

Figure S7

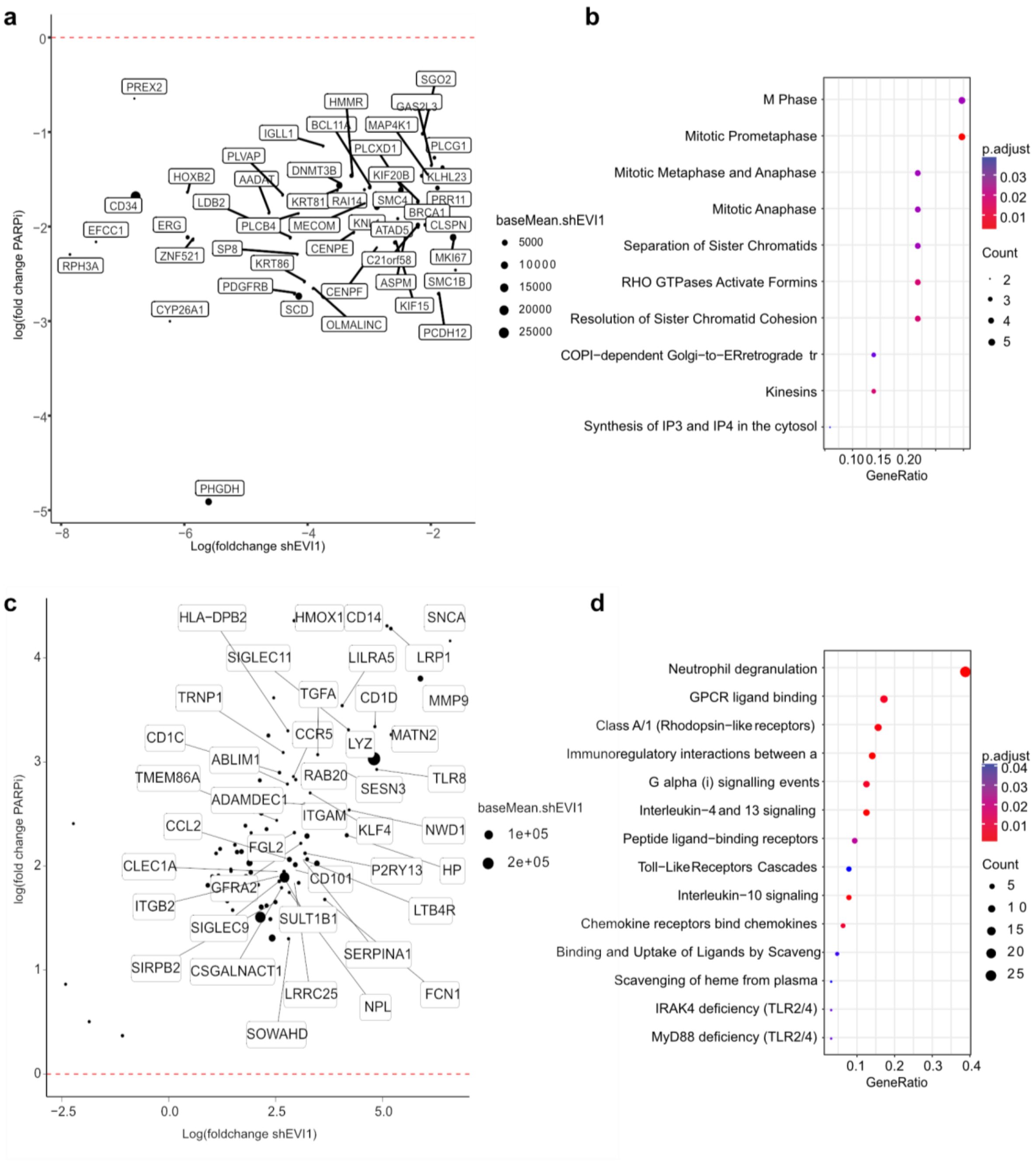

a
b
c
d
PREX2
SGO2
HMMR
GAS2L3
BCL11A
MAP4K1
IGLL1
PLCXD1
PLCG1
PLVAP
DNMT3B
KIF20B
KLHL23
HOXB2
AADAT
KRT81
RAI14
SMC4
PRR11
CD34
LDB2
BRCA1
ERG
PLCB4
MECOM
KNL1
ATAD5
CLSPN
EFCC1
ZNF521
SP8
CENPE
C21orf58
MKI67
RPH3A
KRT86
ASPM
SMC1B
PDGFRB
CENPF
KIF15
CYP26A1
SCD
OLMALINC
PCDH12
PHGDH
log(fold change PARPi)
Log(foldchange shEVI1)
baseMean.shEVI1
5000
10000
15000
20000
25000
M Phase
Mitotic Prometaphase
Mitotic Metaphase and Anaphase
Mitotic Anaphase
Separation of Sister Chromatids
RHO GTPases Activate Formins
Resolution of Sister Chromatid Cohesion
COPI−dependent Golgi−to−ERretrograde tr
Kinesins
Synthesis of IP3 and IP4 in the cytosol
p.adjust
0.03
0.02
0.01
Count
2
3
4
5
GeneRatio
HLA−DPB2
HMOX1
CD14
SNCA
SIGLEC11
LILRA5
LRP1
TRNP1
TGFA
CD1D
MMP9
CD1C
CCR5
LYZ
MATN2
ABLIM1
TMEM86A
RAB20
TLR8
SESN3
ADAMDEC1
ITGAM
CCL2
KLF4
NWD1
FGL2
CLEC1A
P2RY13
HP
GFRA2
CD101
ITGB2
LTB4R
SIGLEC9
SULT1B1
SERPINA1
SIRPB2
CSGALNACT1
LRRC25
FCN1
NPL
SOWAHD
1e+05
2e+05
Neutrophil degranulation
GPCR ligand binding
Class A/1 (Rhodopsin−like receptors)
Immunoregulatory interactions between a
G alpha (i) signalling events
Interleukin−4 and 13 signaling
Peptide ligand−binding receptors
Toll−Like Receptors Cascades
Interleukin−10 signaling
Chemokine receptors bind chemokines
Binding and Uptake of Ligands by Scaveng
Scavenging of heme from plasma
IRAK4 deficiency (TLR2/4)
MyD88 deficiency (TLR2/4)
0.04
10
15
20
25

Supplemental files

The supplementary File S1 “ChIP-SICAP results.xlsx” summarizes the proteins identified by ChIP-SICAP using CEBPA and RUNX1 as baits in the cell lines MUTZ-3, MOLM-1 and HNT-34. Given are the official gene names and Uniprot IDs, as well as the intensity ratios of Bait/IgG for both replicates, the average log of the intensity ratios, the average log of the iBAQ intensities, the p-values and the adjusted p-values (p.adj.).

The supplementary File S2 “TPM values.xlsx” summarizes the normalized transcript per million (TPM) values of *EVI1* and *PARP1* transcripts from RNA-Seq data of primary human AML patient samples (n=73) and cell lines (n=9) and states their karyotype. The occurrence of inv(3)/t(3;3) is marked in an additional column.

The supplementary File S3 “diffExprGenes.xlsx” contains the RNA-Seq results and summarizes the differentially expressed genes after 48 h PARPi treatment or *EVI1* knockdown, respectively. Shown are the mean expression value (baseMean), the gene expression foldchange over the control (DMSO in case of PARPi and non-targeting control in case of *EVI1* Knockdown) as well as the p-values and adjusted p-values.
